# Supplementary figures and images for: Molecular characterization of the permanent outer-inner membrane contact site of the mitochondrial genome segregation complex in trypanosomes
Source: PLoS Pathog. 2024 Dec 2;20(12):e1012635. doi: 10.1371/journal.ppat.1012635 (PMC11637284; doi:10.1371/journal.ppat.1012635)

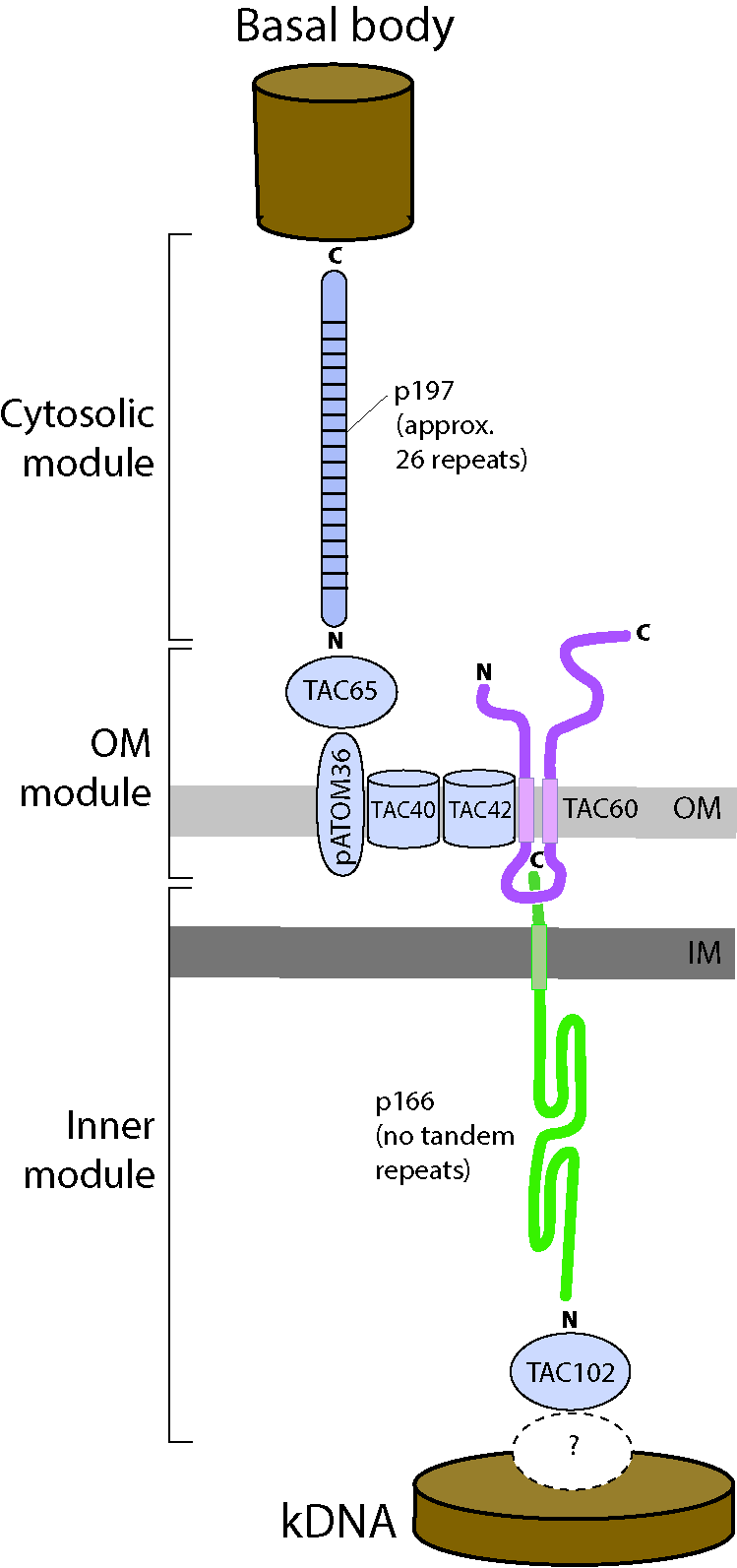

Supplement: S1 Fig — Single unit TAC cable connecting the basal body to the kinetoplast DNA (kDNA). The three molecular TAC modules and the individual TAC subunits are indicated. The TAC consists of several hundreds of TAC cables arranged in a parallel manner. OM, outer membrane; IM, inner membrane. (TIF) [file ppat.1012635.s001.tif]

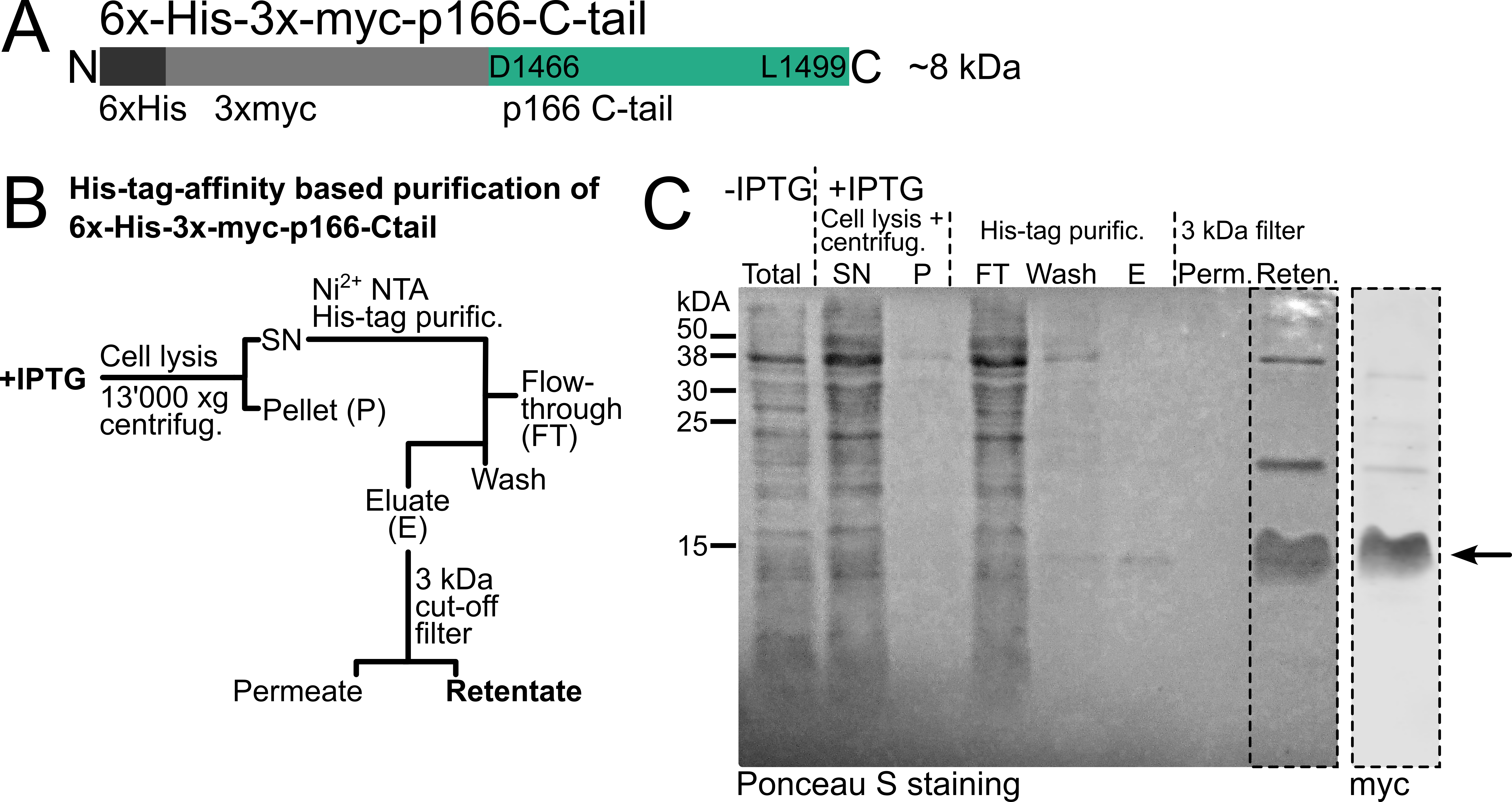

Supplement: S2 Fig — (A) Depiction of the intermembrane space exposed C-terminus of p166 (D1466-L1499) that was N-terminally fused to 6x His and 3x myc tags and recombinantly expressed in E. coli. (B) Workflow for the His-tag affinity purification of the fusionprotein. (C) Left, Ponceau S stain of a blotted SDS-gel monitoring the purification of the recombinant fusion protein. Right, immunoblot staining of the purified protein fraction (Reten.) using an anti-myc antiserum. (TIF) [file ppat.1012635.s002.tif]

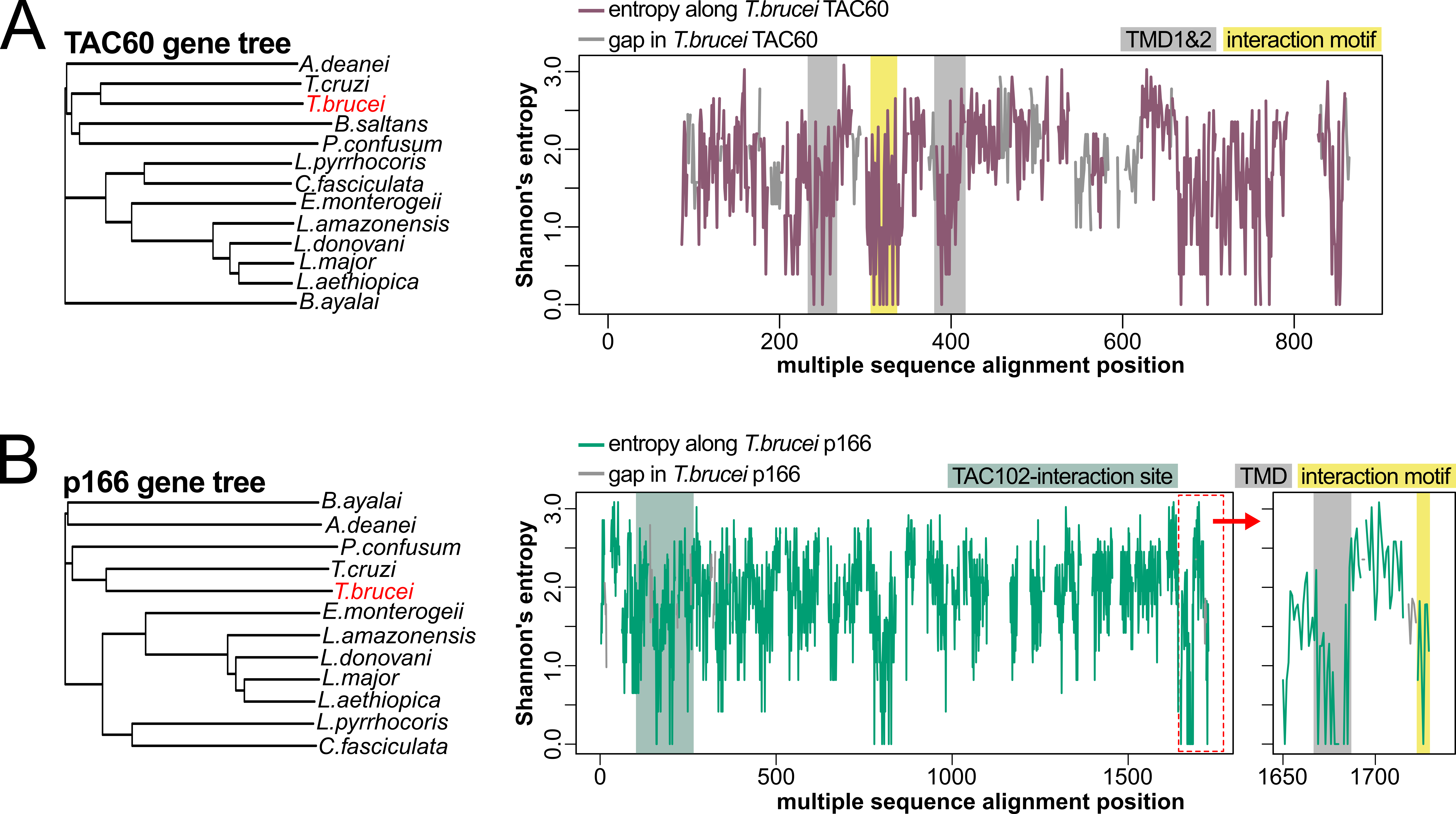

Supplement: S3 Fig — (A) Multiple sequence alignment of TAC60 orthologues from the Kinetoplastid species shown in the phylogenetic tree on the left was analyzed using a Shannon’s entropy plot. (B) Multiple sequence alignment of p166 orthologues from the Kinetoplastid species shown in the phylogenetic tree on the left was analyzed using a Shannon’s entropy plot. The right graph shows a magnification of the C-terminal p166 region depicted by the dashed red line. (TIF) [file ppat.1012635.s003.tif]

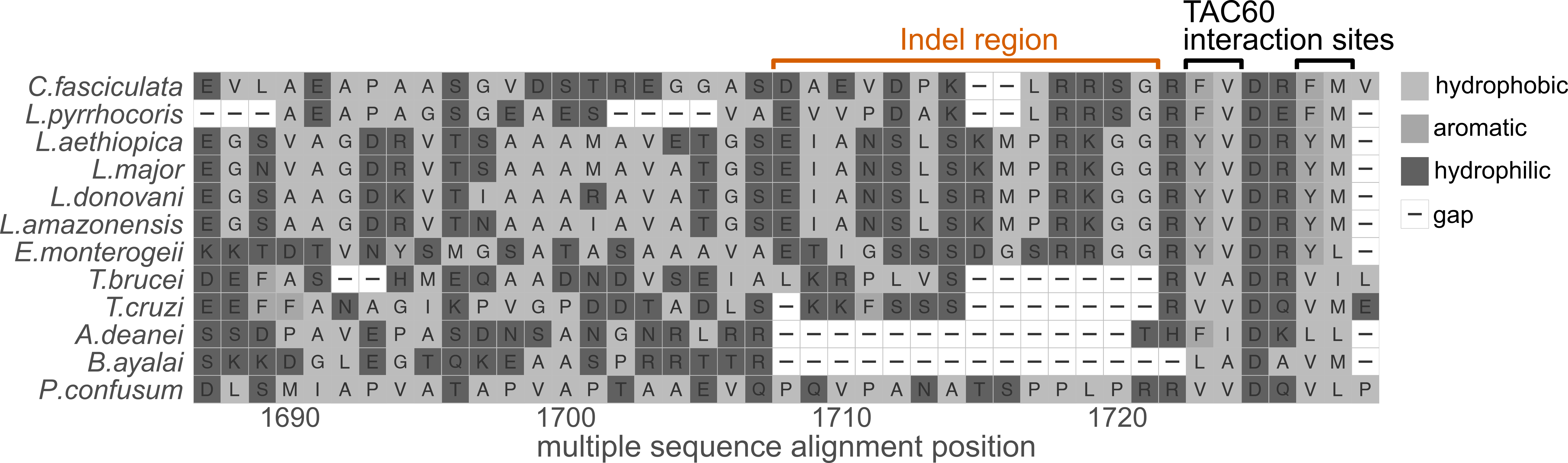

Supplement: S4 Fig — Sequence alignment of the C-terminal region of p166 orthologues of the same Kinetoplastid species as were used for Fig 3B. (TIF) [file ppat.1012635.s004.tif]

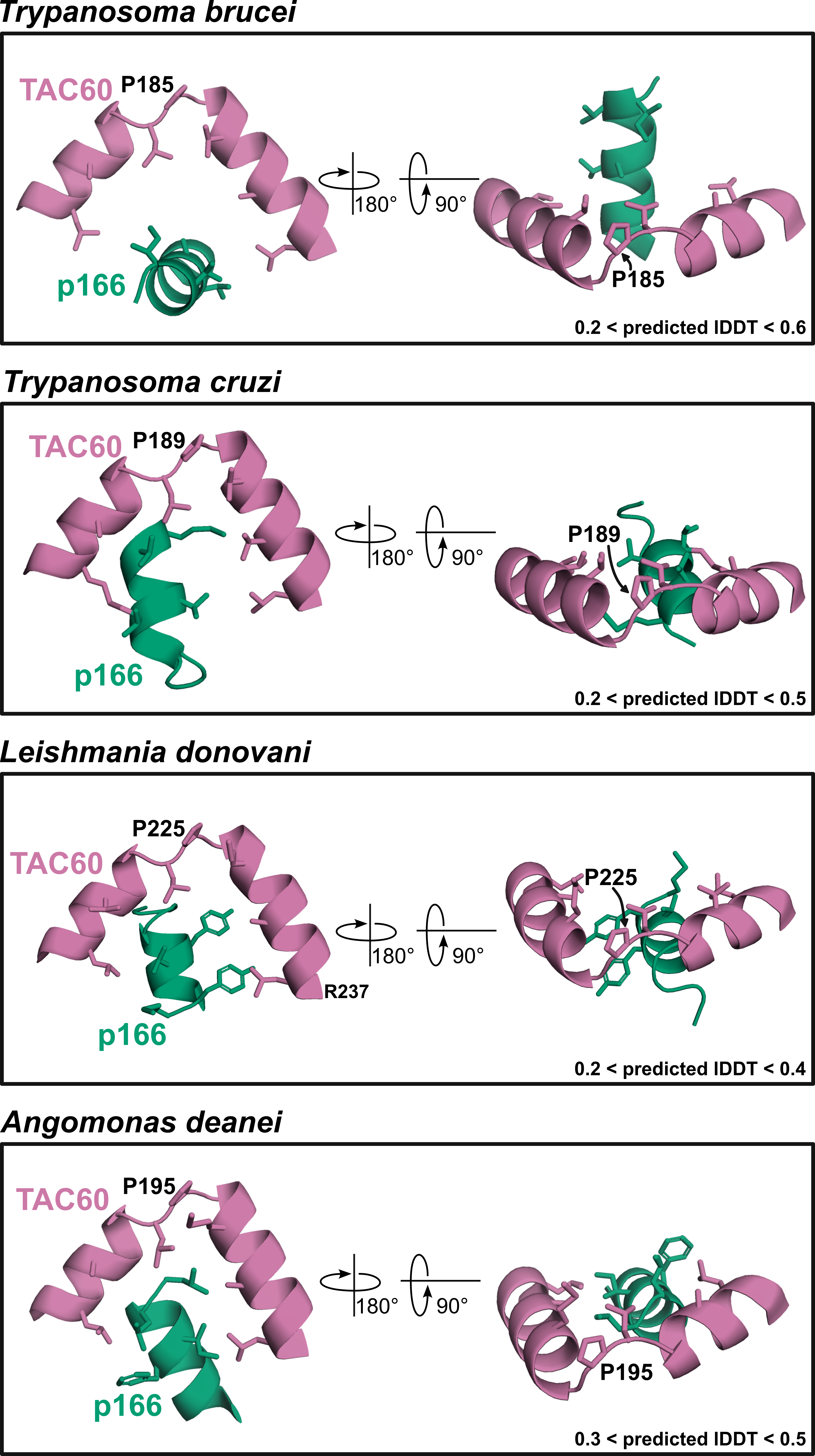

Supplement: S5 Fig — Models depicting the predicted TAC60- p166 interaction interface in T. brucei, T. cruzi, L. donovani, and A. deanei. The predicted structure for T. brucei is identical to the model shown in Fig 1. For the predictions in the other species the following input sequence segments were used: T. cruzi TAC60 (1–233 aa), p166 (1349–1384 aa); L. donovani TAC60 (1–312 aa), p166 (1160–1204 aa); A. deanei TAC60 (1–239 aa), p166 (986–1017 aa). The models display the conserved kinked α-helix of TAC60 beginning 10 aa upstream and ending 12 aa downstream of the conserved P (see Fig 3). The sidechains of the conserved hydrophobic aa are shown as sticks. While the predicted local Distance Difference Test (IDDT) scores were low for all predictions, a hydrophobic pocket is predicted for all the interaction interfaces. (TIF) [file ppat.1012635.s005.tif]

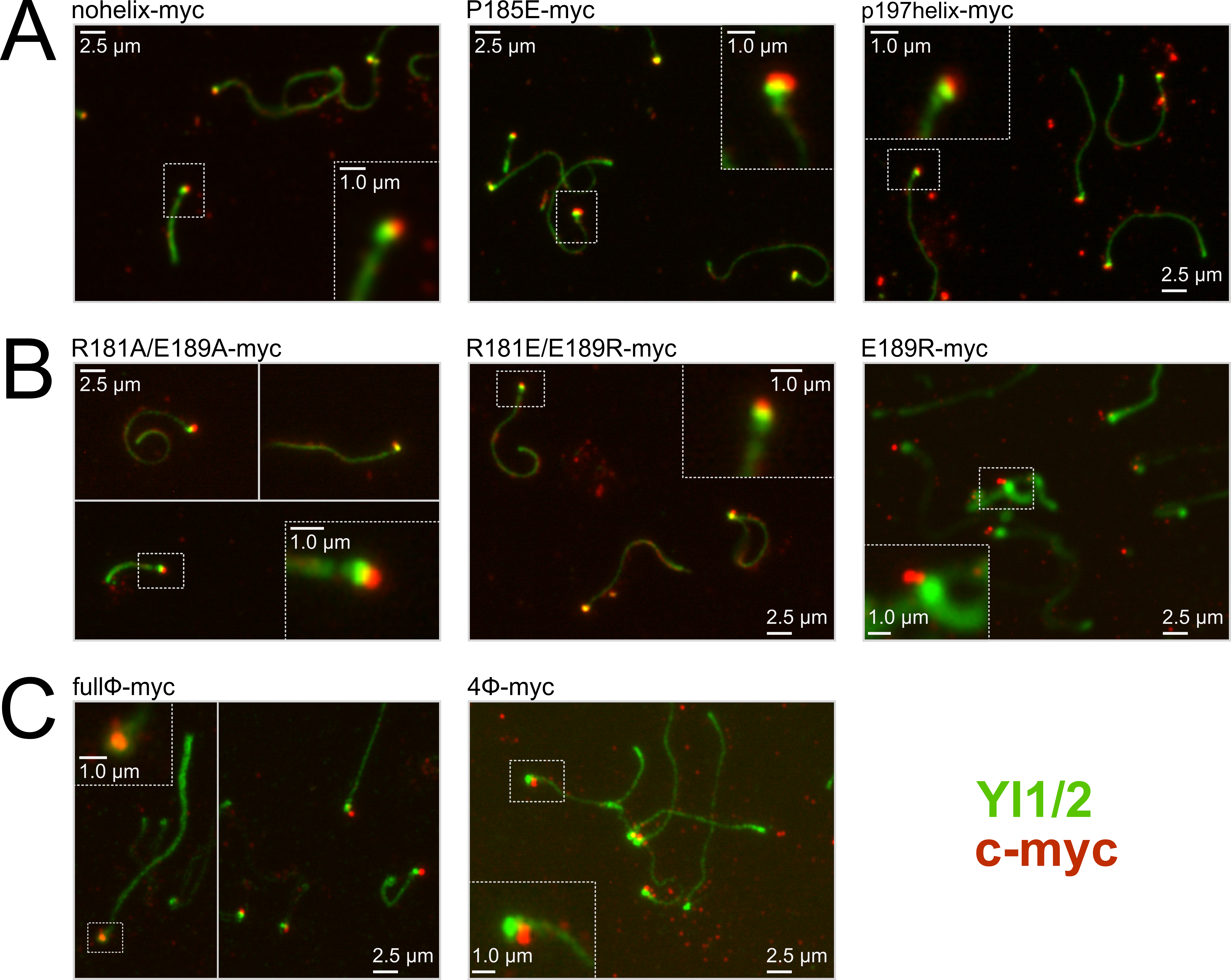

Supplement: S7 Fig — Immunofluorescence images of extracted flagella of the indicated mutant TAC60 cell lines show that the mutant proteins (red) co-fractionate with flagella and colocalize with or very close to the basal body. This indicates that the mutant proteins are integrated into the TAC. Tyrosinated tubulin and TbRP2, detected by YL1/2 (green) serves a marker for the flagellum and basal body. Broken line mark original and enlarged insets. (TIF) [file ppat.1012635.s007.tif]
