# Supplementary material for: Molecular characterization of the permanent outer-inner membrane contact site of the mitochondrial genome segregation complex in trypanosomes
Source: PLoS Pathog. 2024 Dec 2;20(12):e1012635. doi: 10.1371/journal.ppat.1012635 (PMC11637284; doi:10.1371/journal.ppat.1012635)
Supplement: S1 Table — The control experiments that the TAC60 mutants used in our study completely replace the endogenous TAC60 and are fully integrated into the TAC are provided in S6 and S7 Figs, respectively (PDF) [file ppat.1012635.s008.pdf]

	TAC60 variants based on TAC60ΔC283		
wild type		175 198<br>wt EHLQRARLALPSFVEYFAKMLLRL	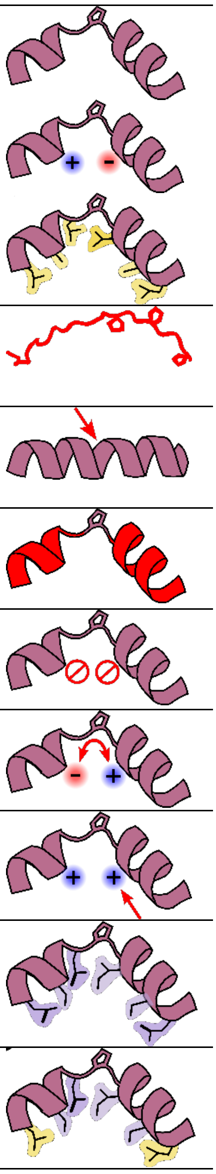
motif folding	TAC60-nohelix	EHLSALQMELIEPTPHILIPLLRL<br>175||| | ||||198<br>wt EHLQRARLALPSFVEYFAKMLLRL	
	TAC60-P185E	EHLQRARLALESFVEYFAKMLLRL<br>175|||||||||| |||||||||||||198<br>wt EHLQRARLALPSFVEYFAKMLLRL	
	TAC60-p197helix	EHLAMHAAEEPERAMHAAEELLRL<br>175||| | | |||198<br>wt EHLQRARLALPSFVEYFAKMLLRL	
conserved charged aa	TAC60-R181A/E189A	EHLQRAALALPSFVAYFAKMLLRL<br>175|||||| ||||||| |||||||||198<br>wt EHLQRARLALPSFVEYFAKMLLRL	
	TAC60-R181E/E189R	EHLQRAELALPSFVRYFAKMLLRL<br>175|||||| ||||||| |||||||||198<br>wt EHLQRARLALPSFVEYFAKMLLRL	
	TAC60-E189R	EHLQRARLALPSFVRYFAKMLLRL<br>175|||||||||||||| |||||||||198<br>wt EHLQRARLALPSFVEYFAKMLLRL	
conserved hydrophobic aa	TAC60-fullΦ	EHNQRSRLANPSFTEYFSKMLNRL<br>175|| || ||| ||| ||| ||| ||198<br>wt EHLQRARLALPSFVEYFAKMLLRL	
	TAC60-4Φ	EHLQRSRLANPSFTEYFSKMLLRL<br>175|||| ||| ||| ||| ||||||198<br>wt EHLQRARLALPSFVEYFAKMLLRL	

	p166 variants based on mini-p166		
wild type		1488 1499<br>wt PLVSRVADRVIL	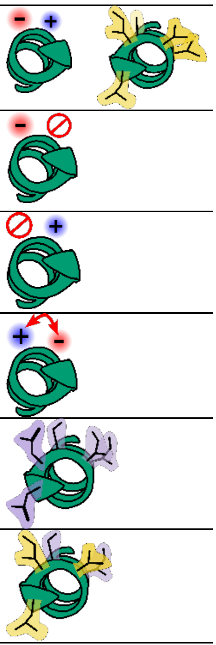
conserved charged aa	p166-R1492A	PLVSAVADRVIL<br>1488|||| |||||||1499<br>wt PLVSRVADRVIL	
	p166-D1495A	PLVSRVAARVIL<br>1488||||||| ||||1499<br>wt PLVSRVADRVIL	
	p166-R1492D/D1495R	PLVSDVARRVIL<br>1488|||| || ||||1499<br>wt PLVSRVADRVIL	
conserved hydrophobic aa	p166-fullΦ	PNTSRTSDRTNN<br>1488| || || 1499<br>wt PLVSRVADRVIL	
	p166-2Φ	PLVSRTSDRVIL<br>1488||||| |||||1499<br>wt PLVSRVADRVIL	
