# Supplementary material for: Molecular characterization of the permanent outer-inner membrane contact site of the mitochondrial genome segregation complex in trypanosomes
Source: PLoS Pathog. 2024 Dec 2;20(12):e1012635. doi: 10.1371/journal.ppat.1012635 (PMC11637284; doi:10.1371/journal.ppat.1012635)

Raw images

Figure 4 B (western blot)

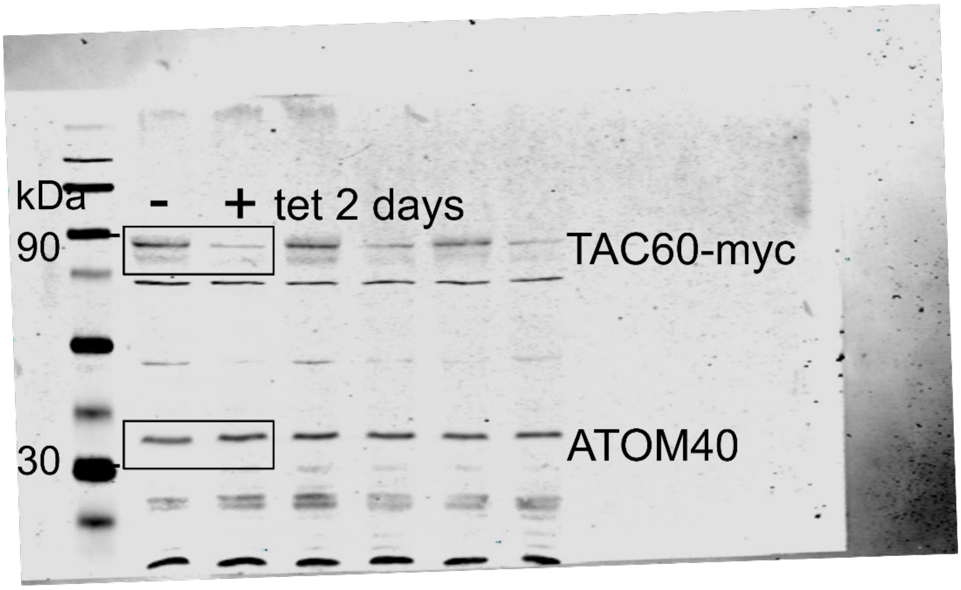

Figure 4 C (western blot)

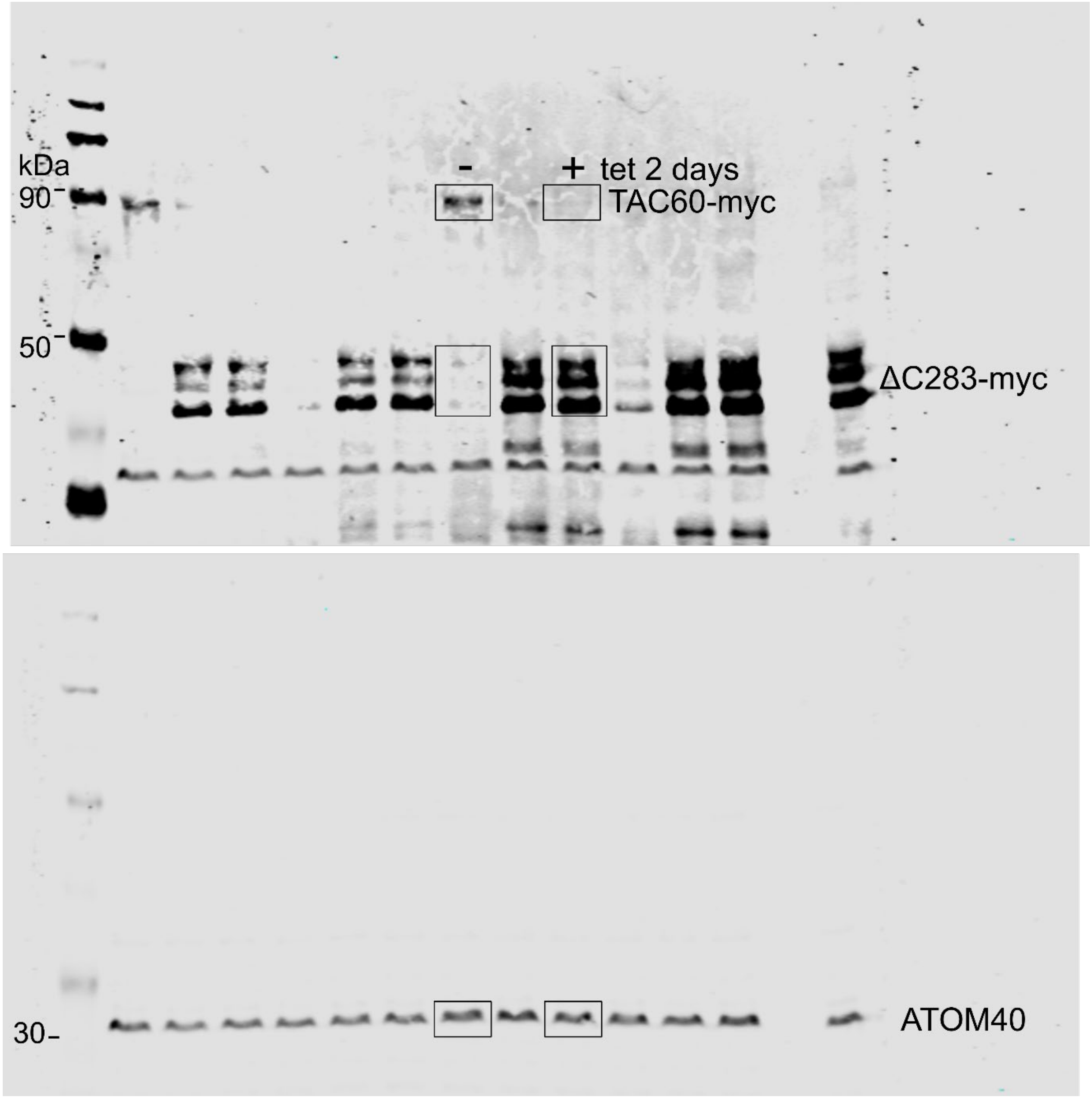

Figure 4 D (western blot)

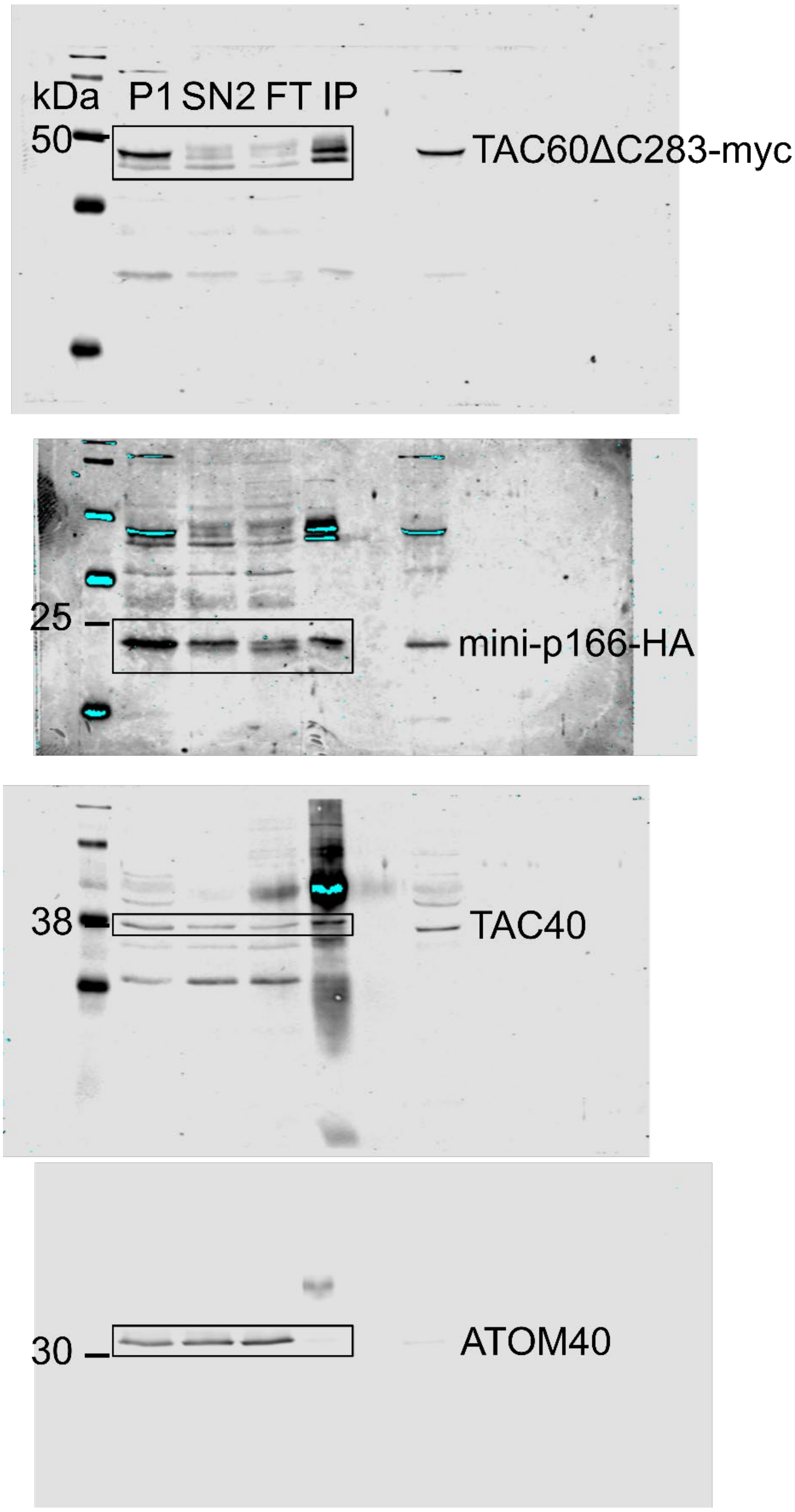

Figure 5 A (western blot)

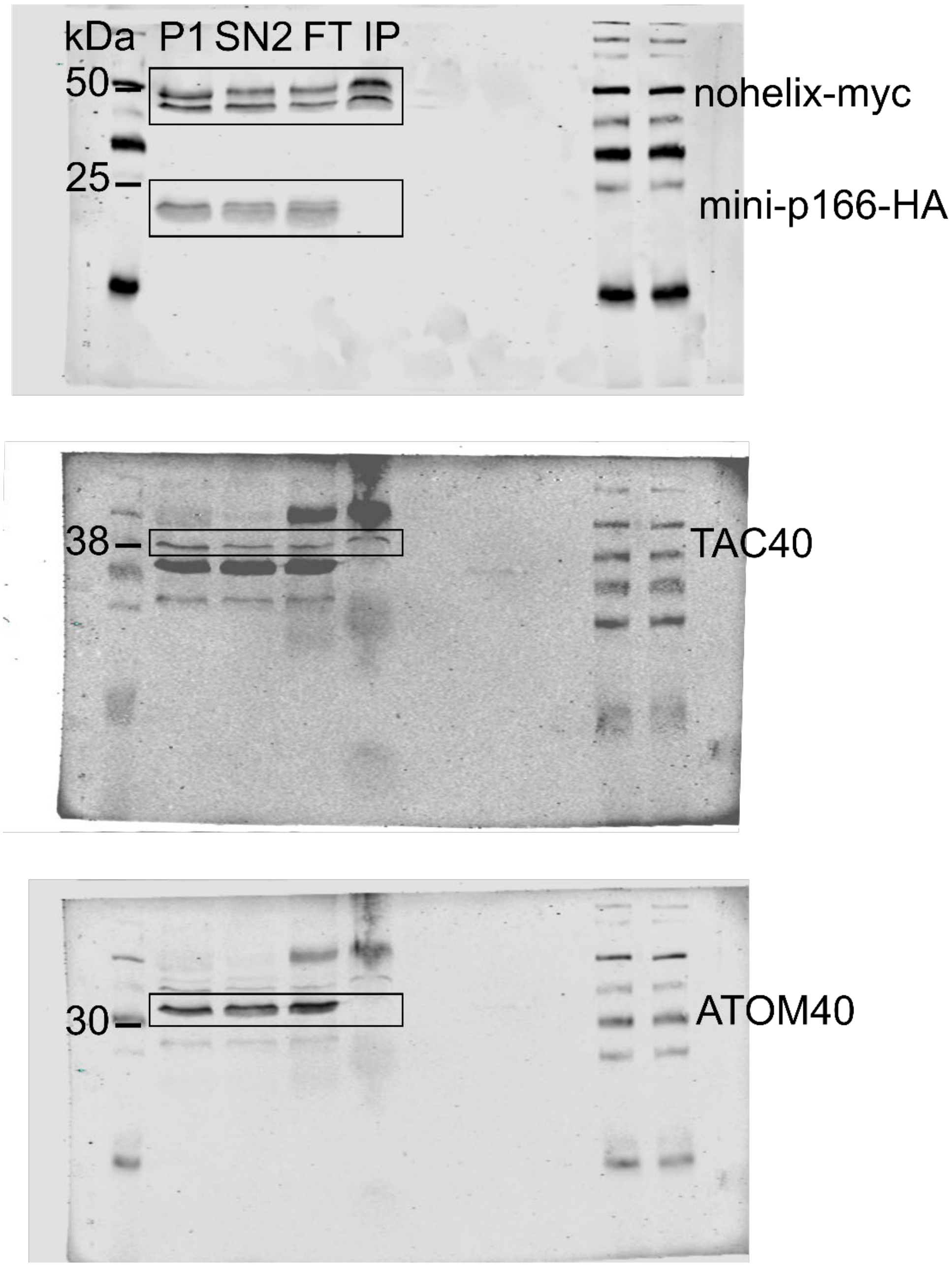

Figure 5 B (western blot)

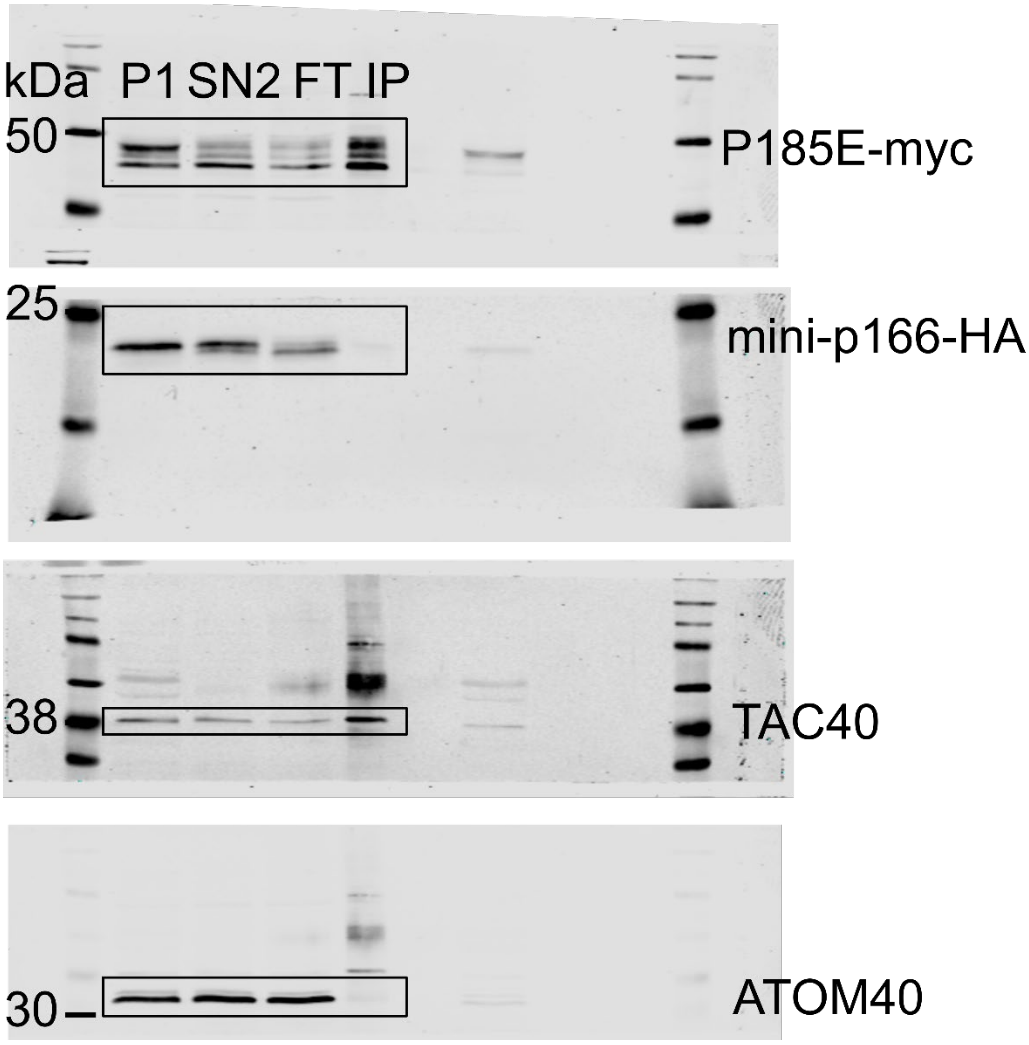

Figure 5 C (western blot)

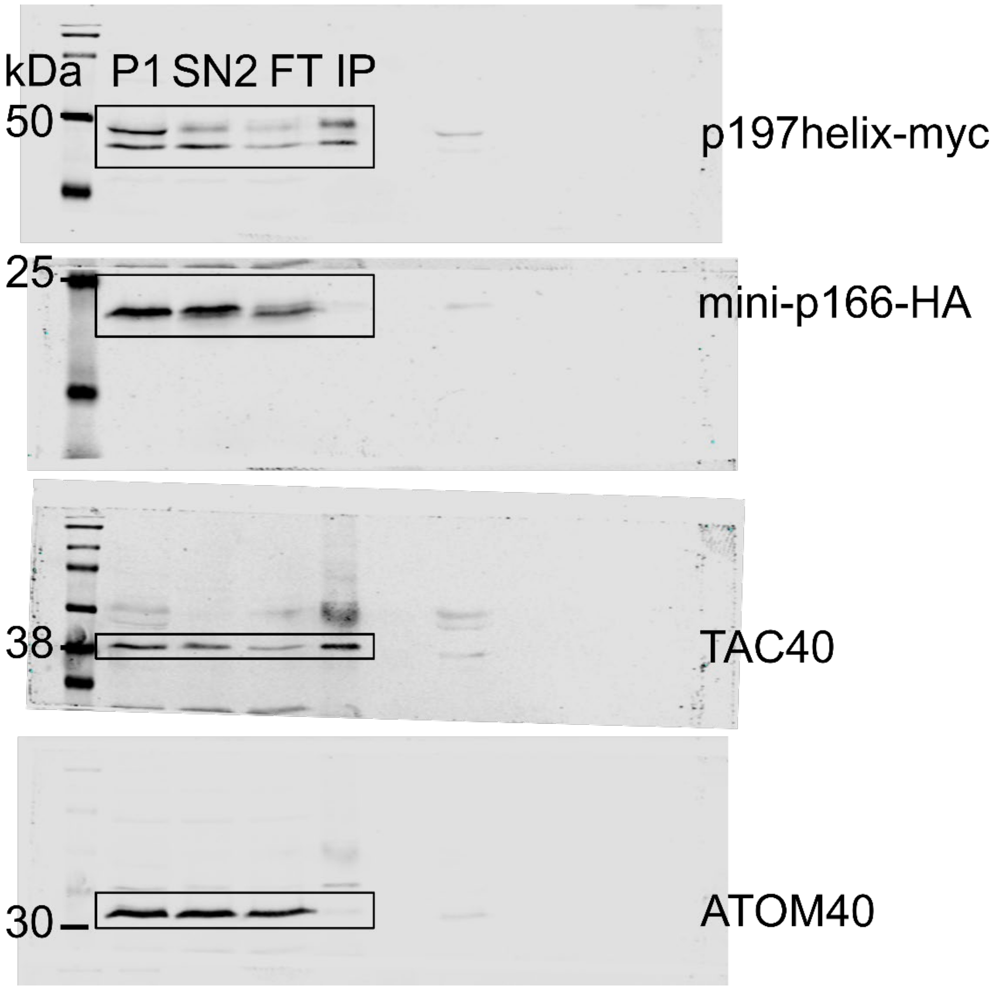

Figure 6 B (western blot)

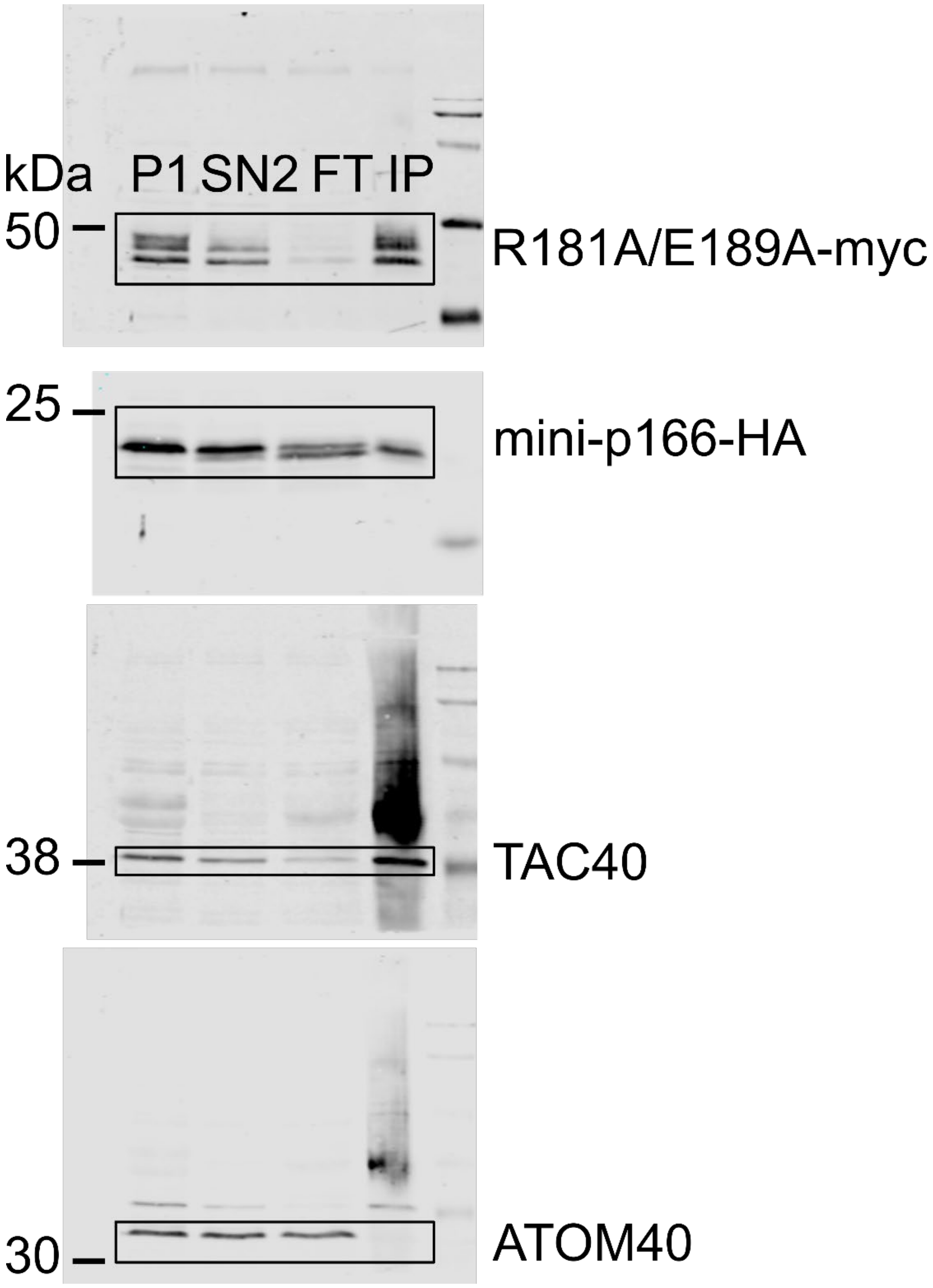

Figure 6 C (western blot)

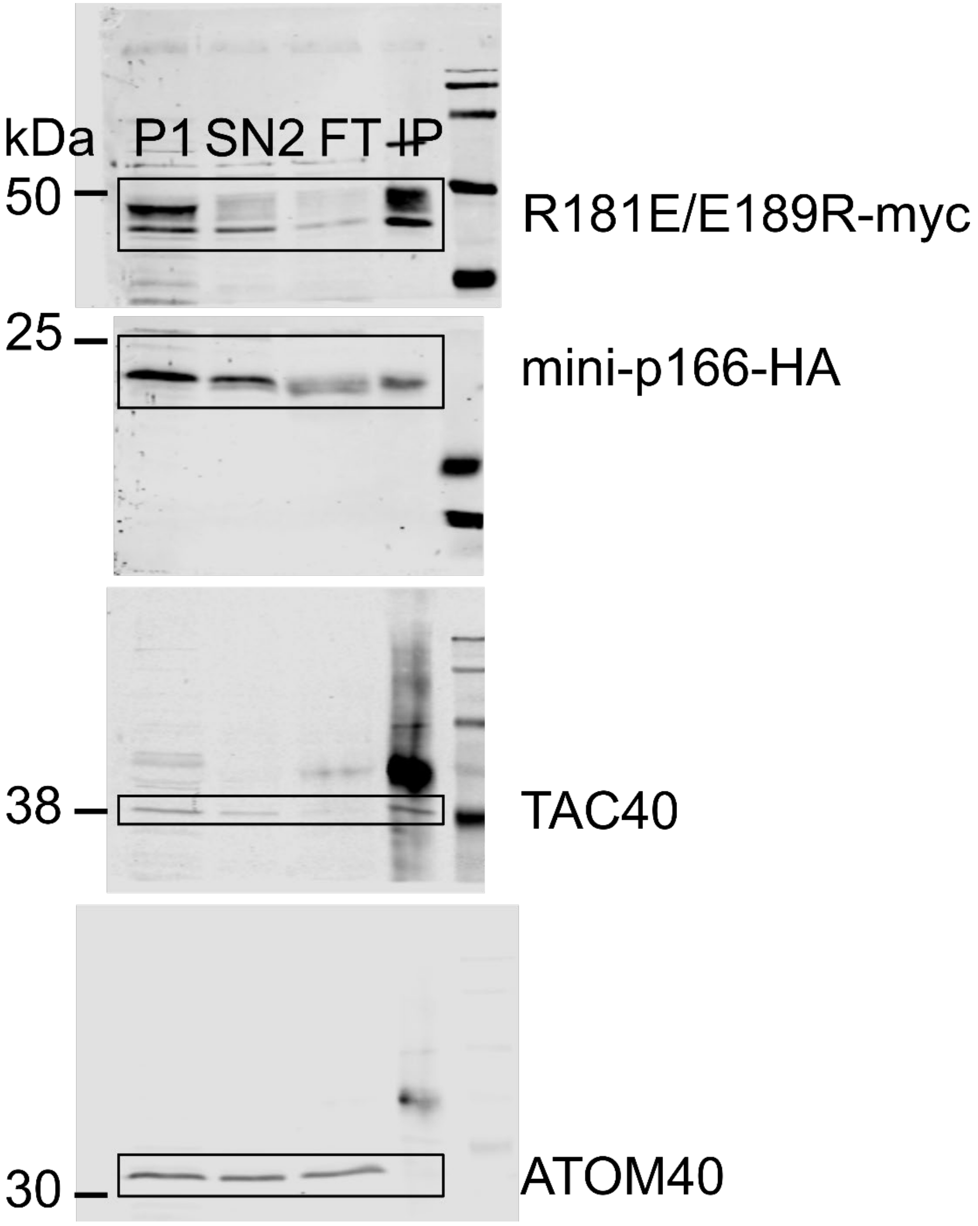

Figure 6 D (western blot)

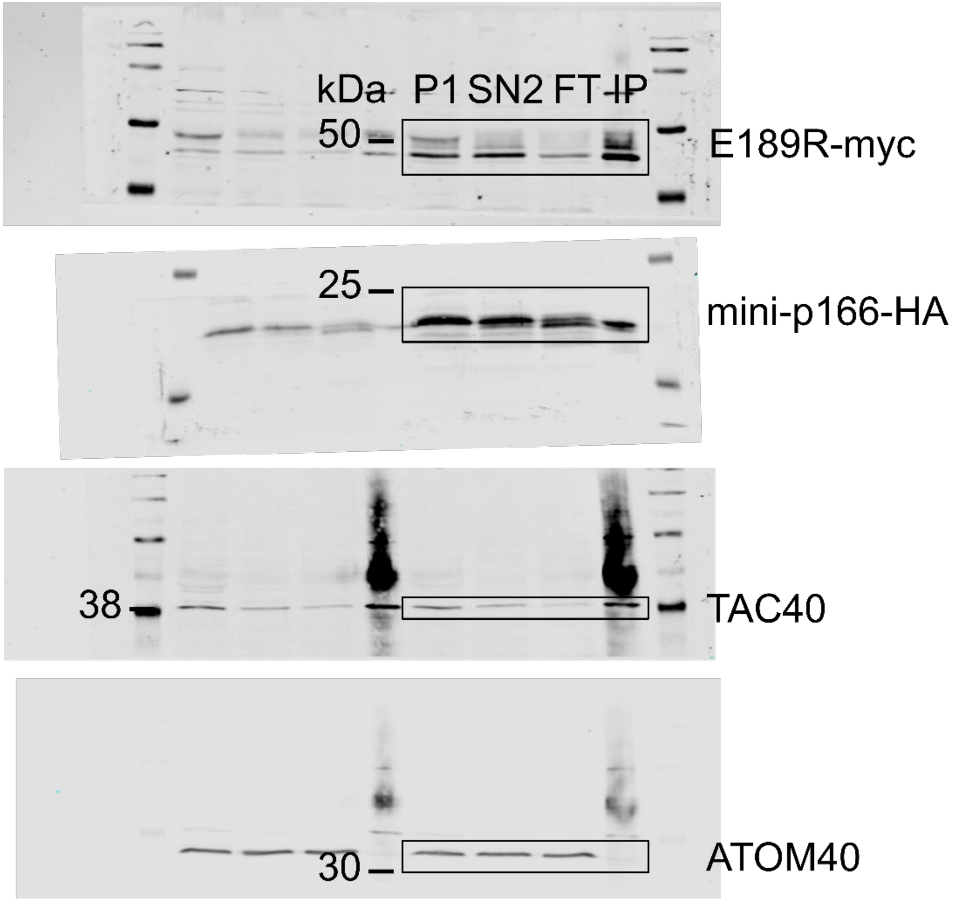

Figure 6 E (western blot)

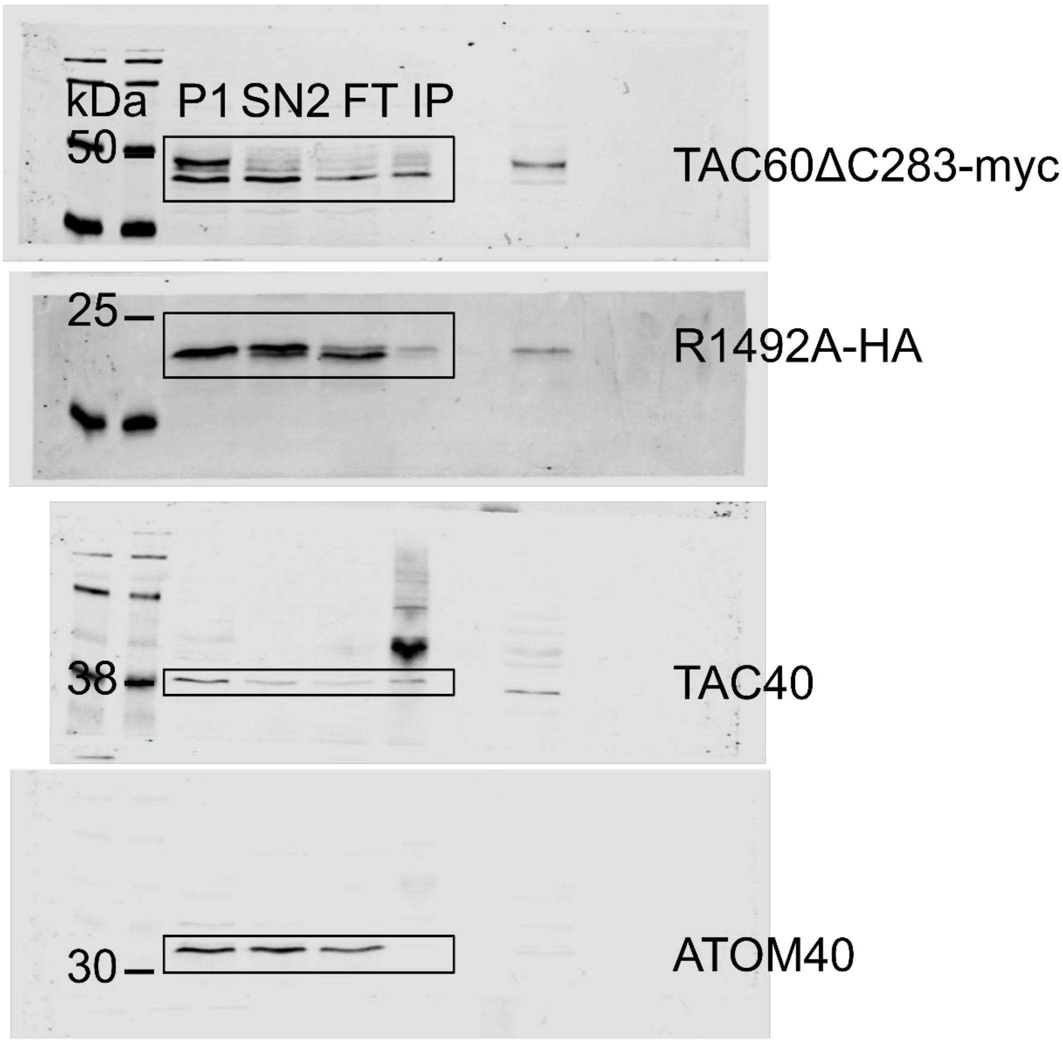

Figure 6 F (western blot)

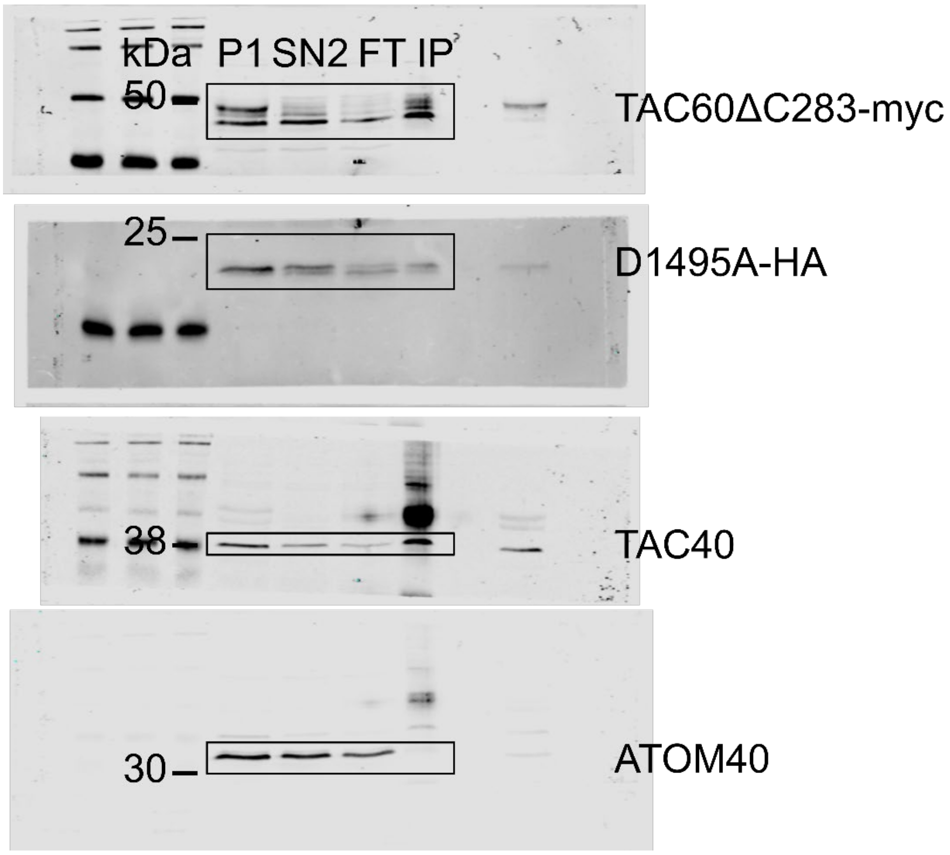

Figure 6 G (western blot)

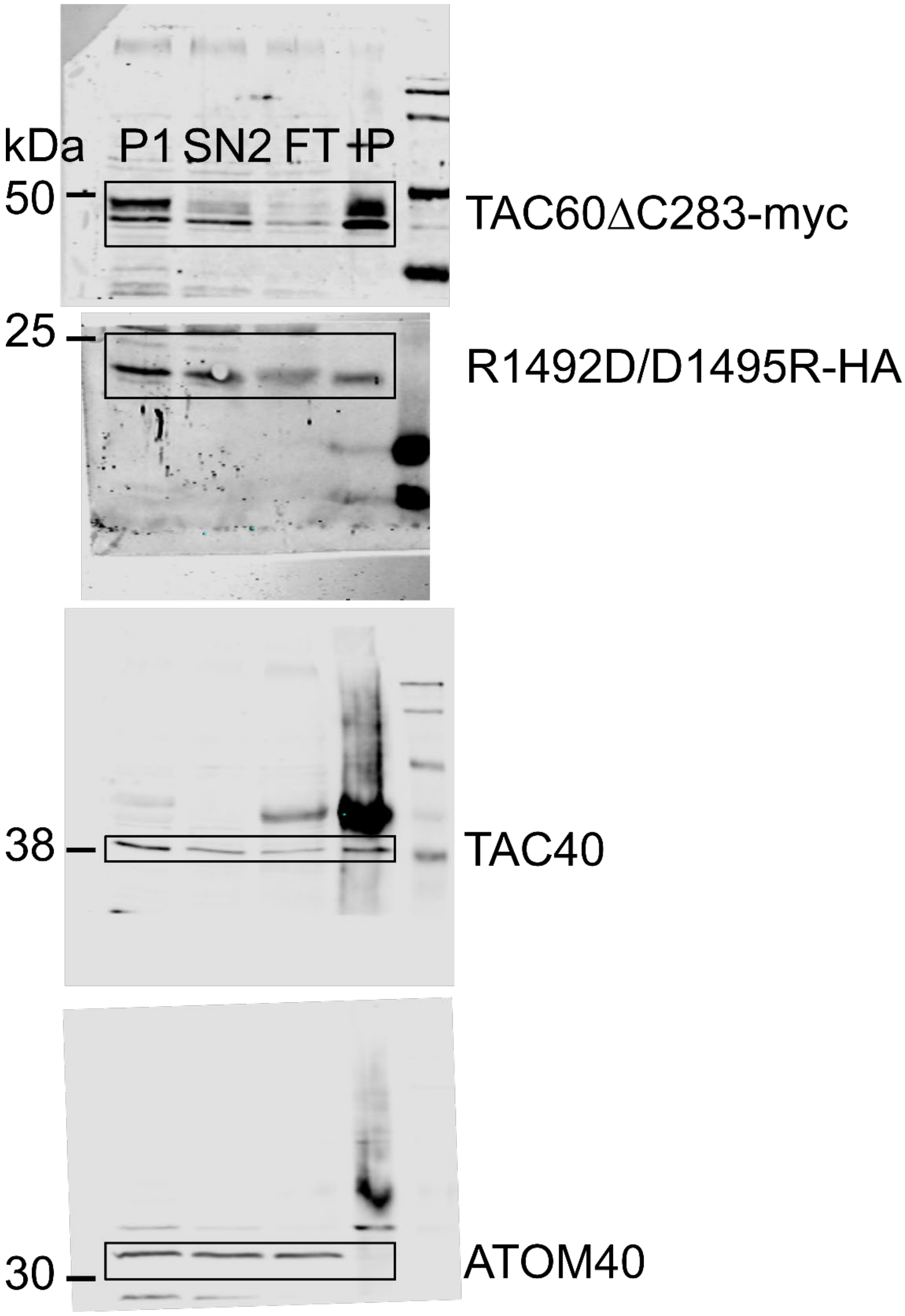

Figure 7 B (western blot)

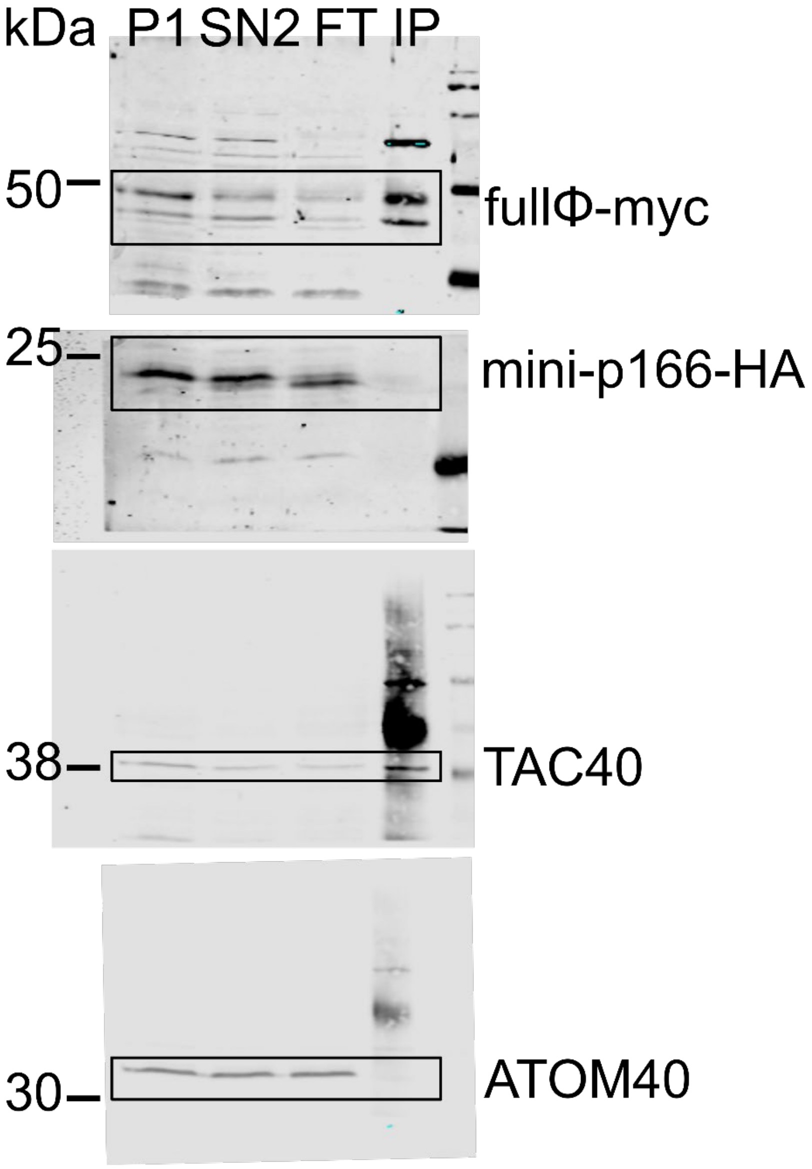

Figure 7 C (western blot)

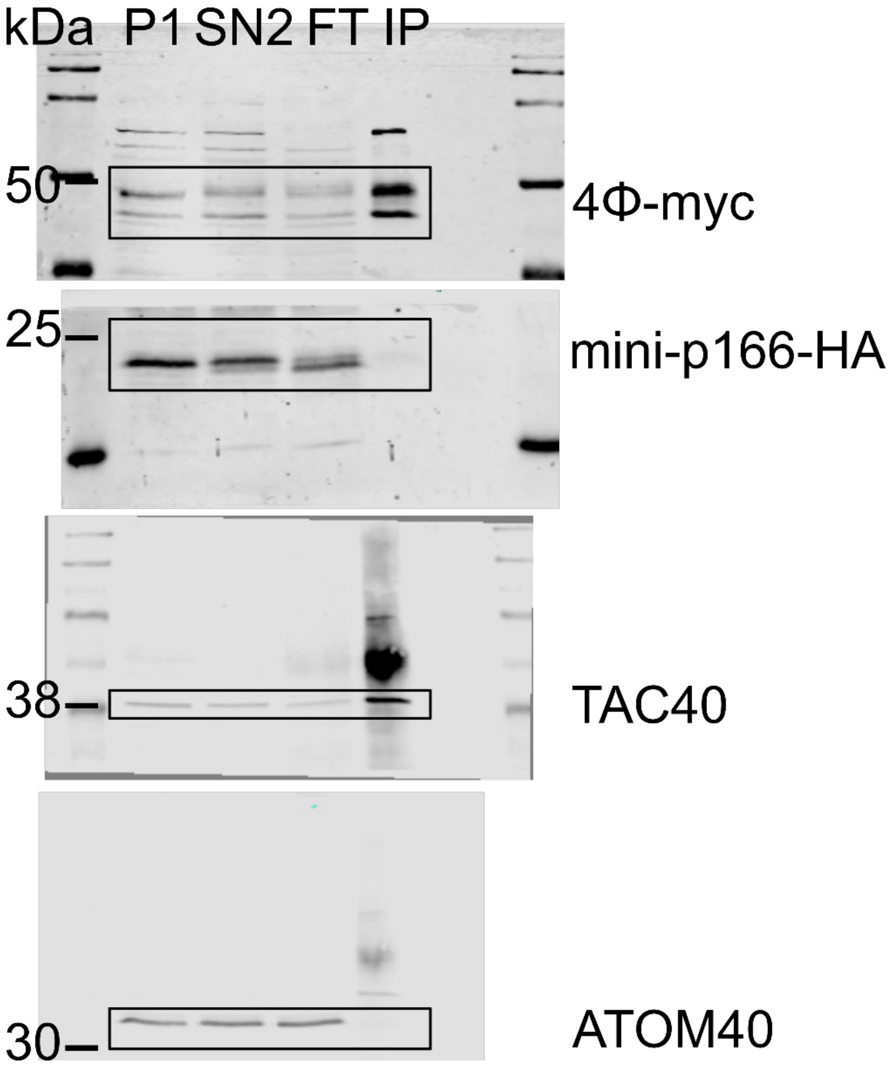

Figure 7 D (western blot)

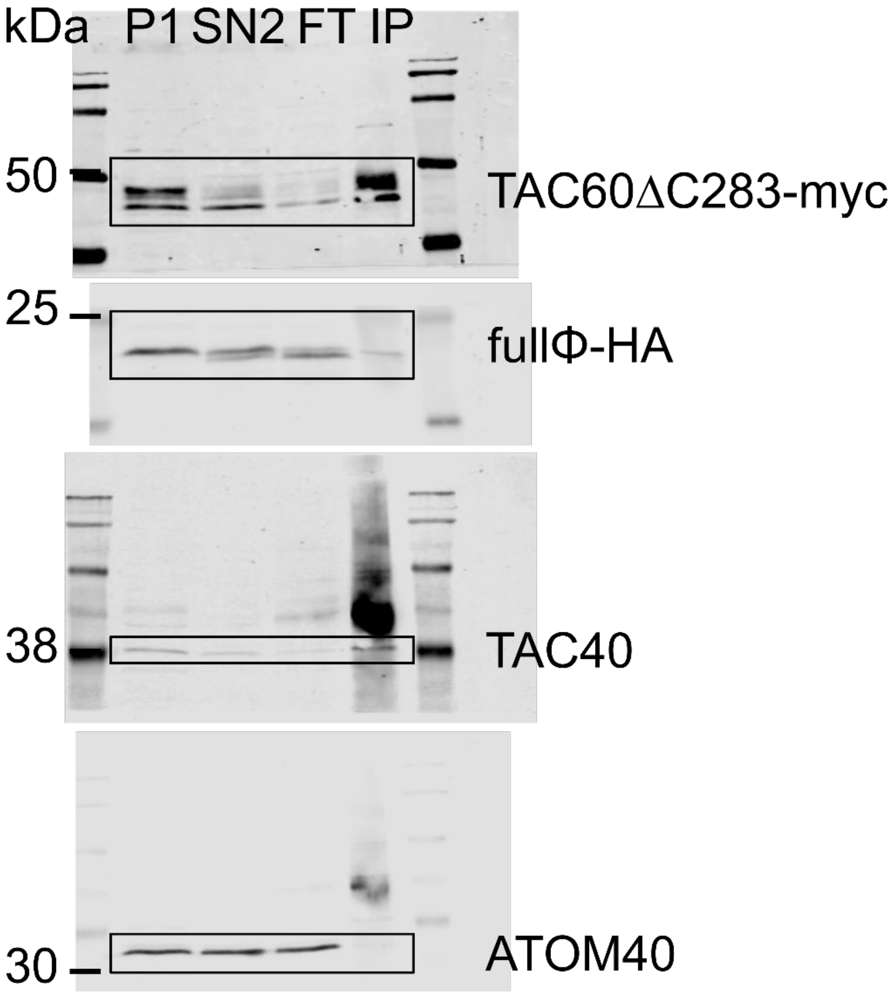

Figure 7 E (western blot)

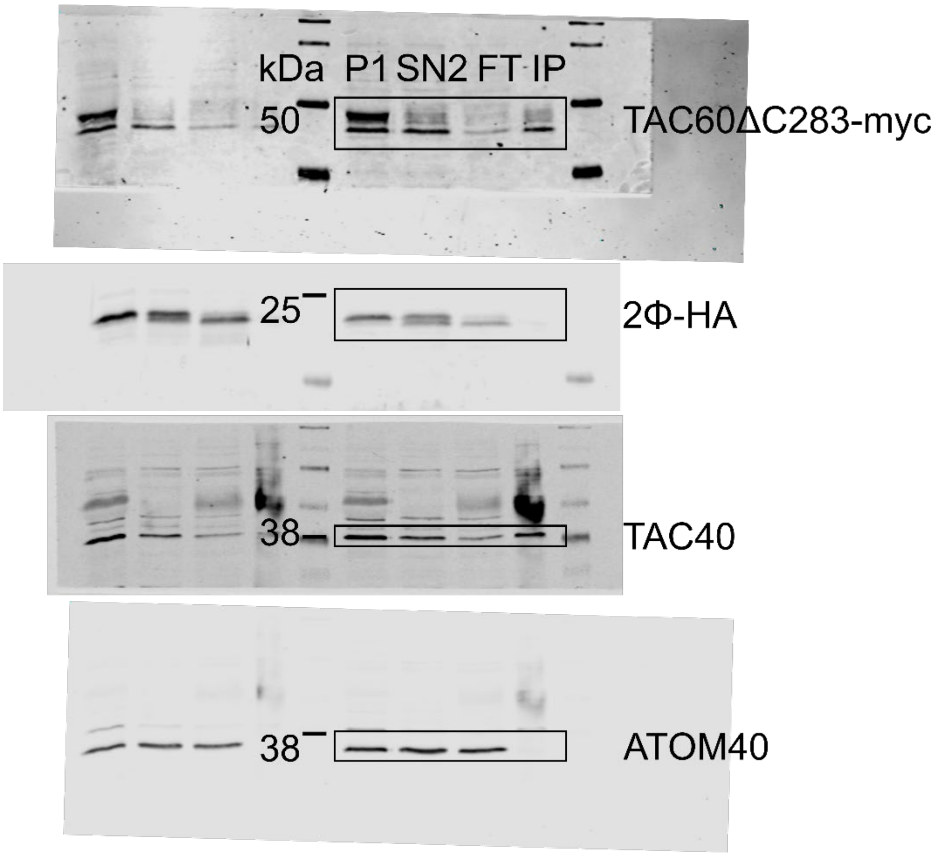

Figure 8 A (western blot)

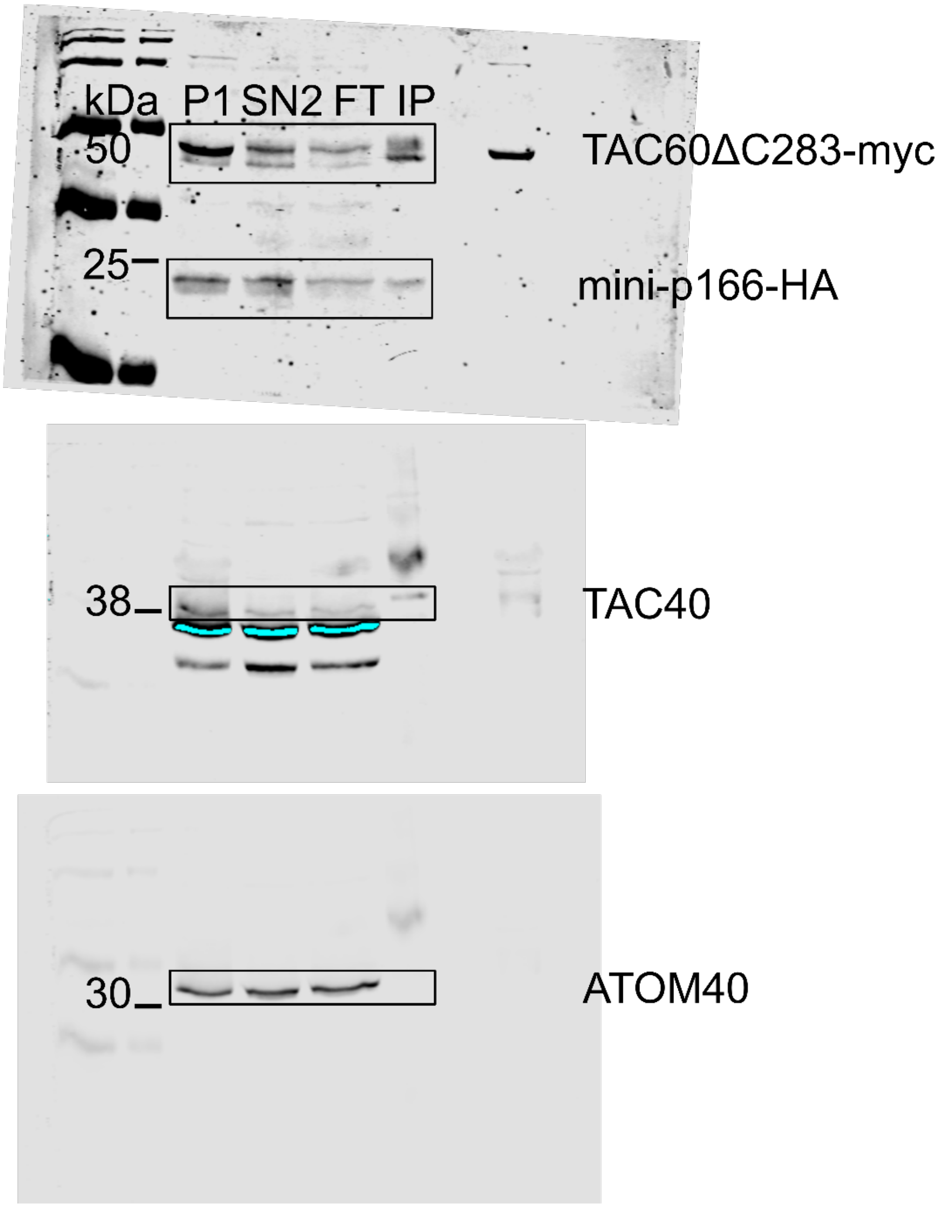

Figure 8 B (western blot)

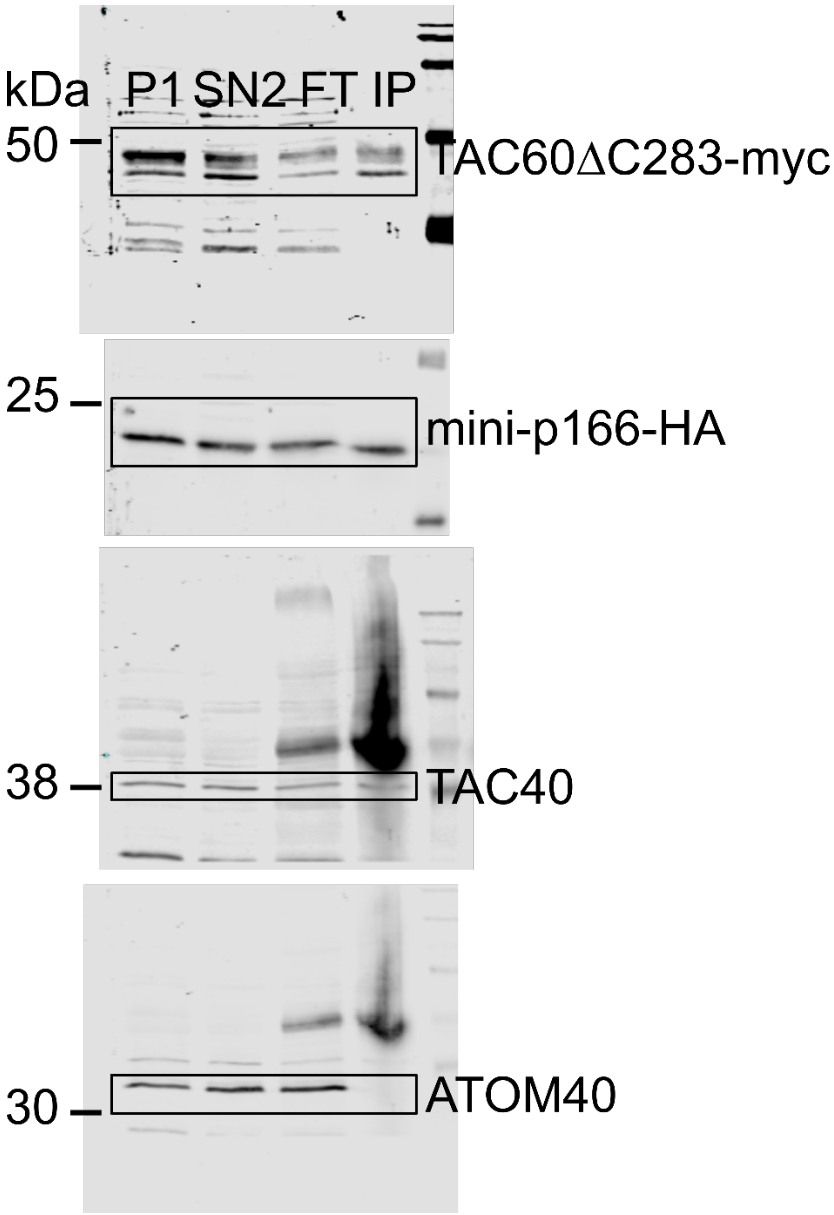

S2 Figure C (western blot)

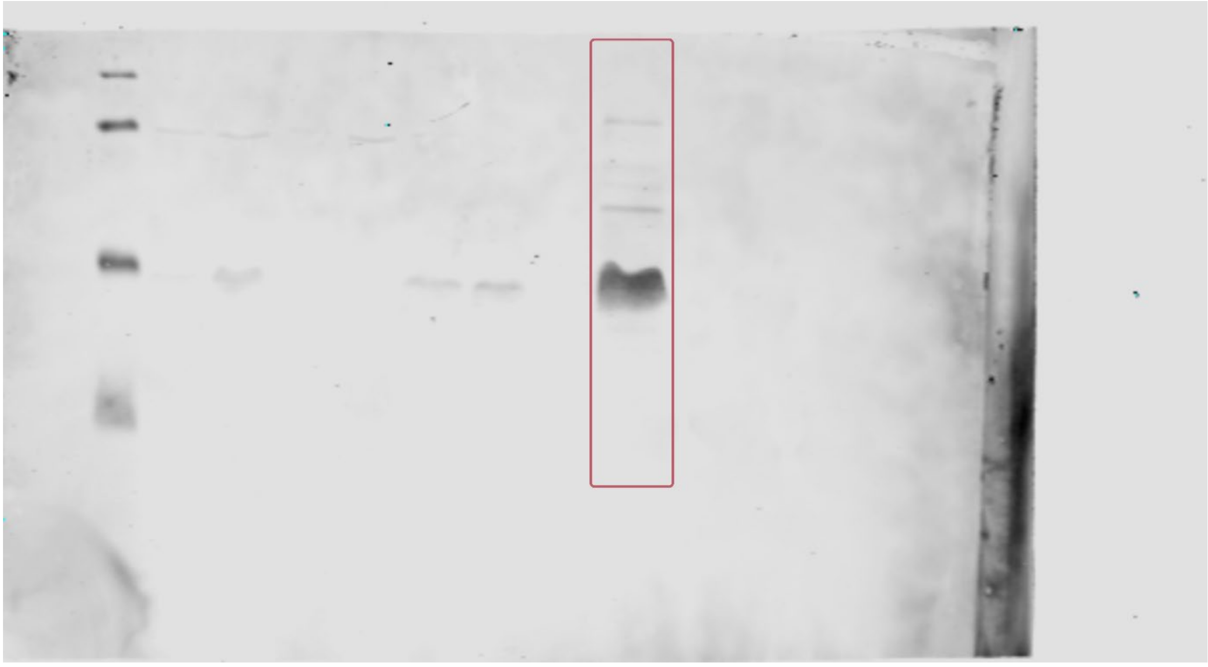

S6 Figure A left (western blot)

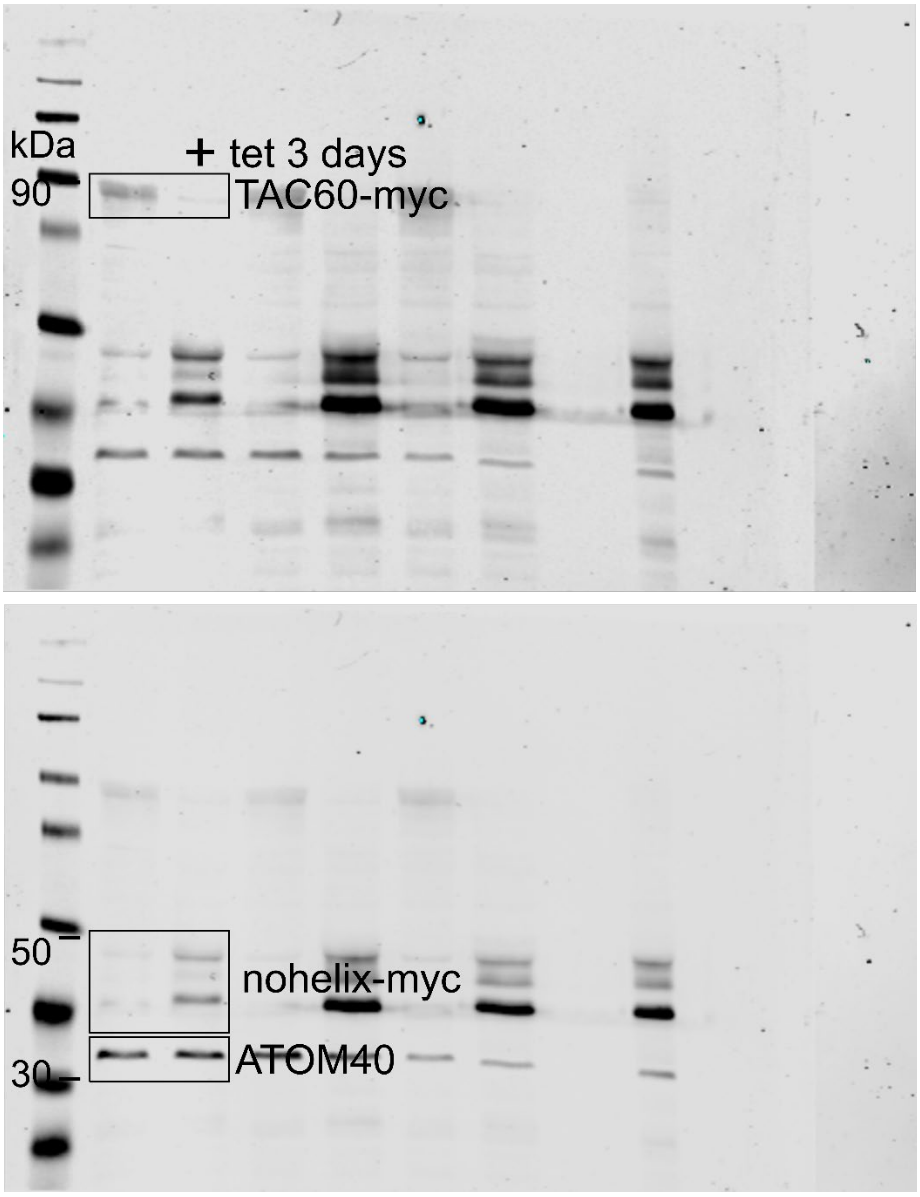

S6 Figure A middle (western blot)

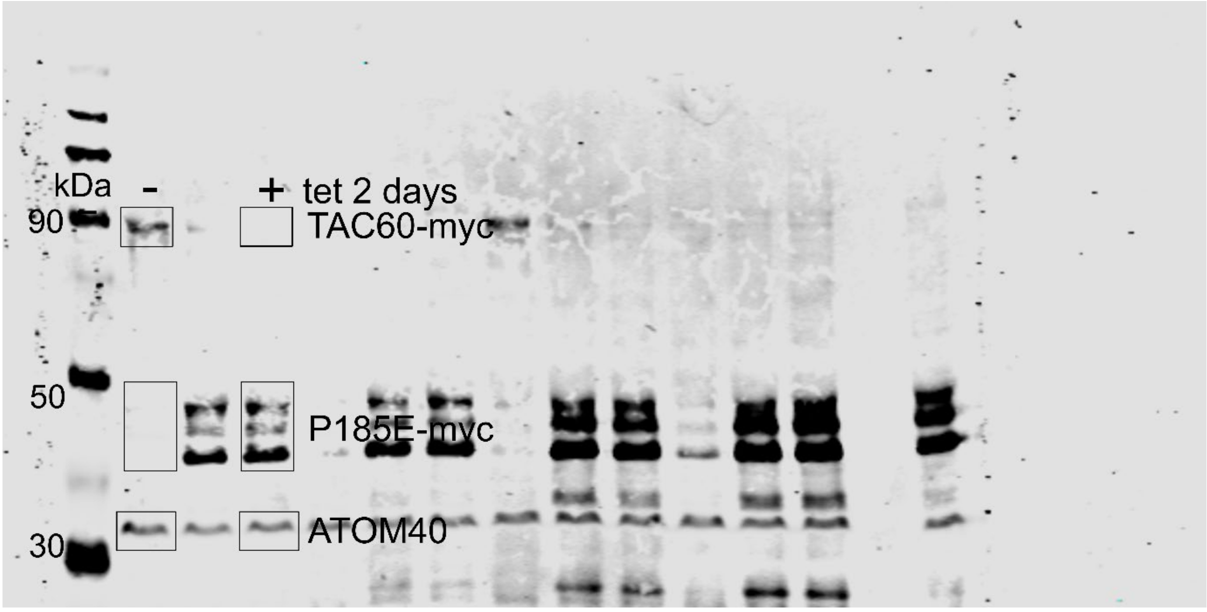

S6 Figure A right (western blot)

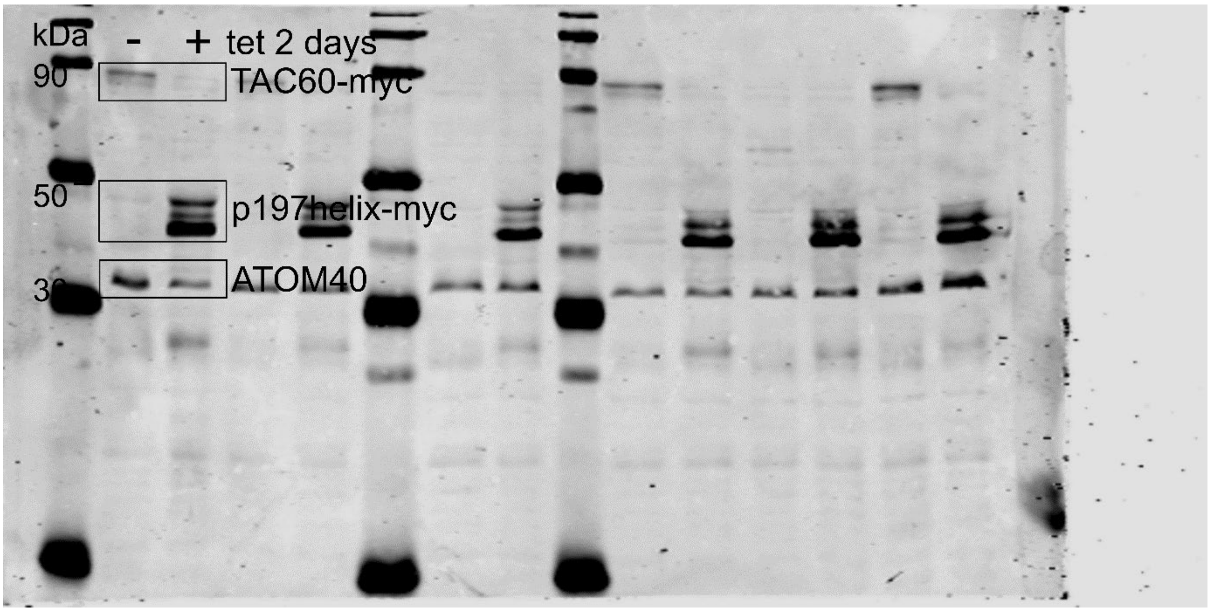

S6 Figure B left (western blot)

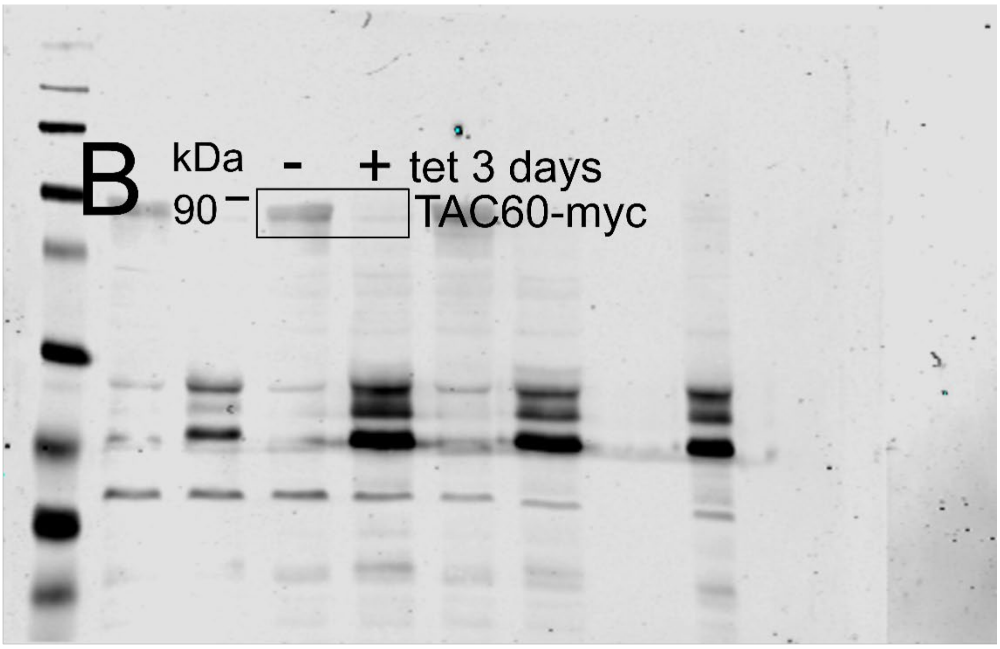

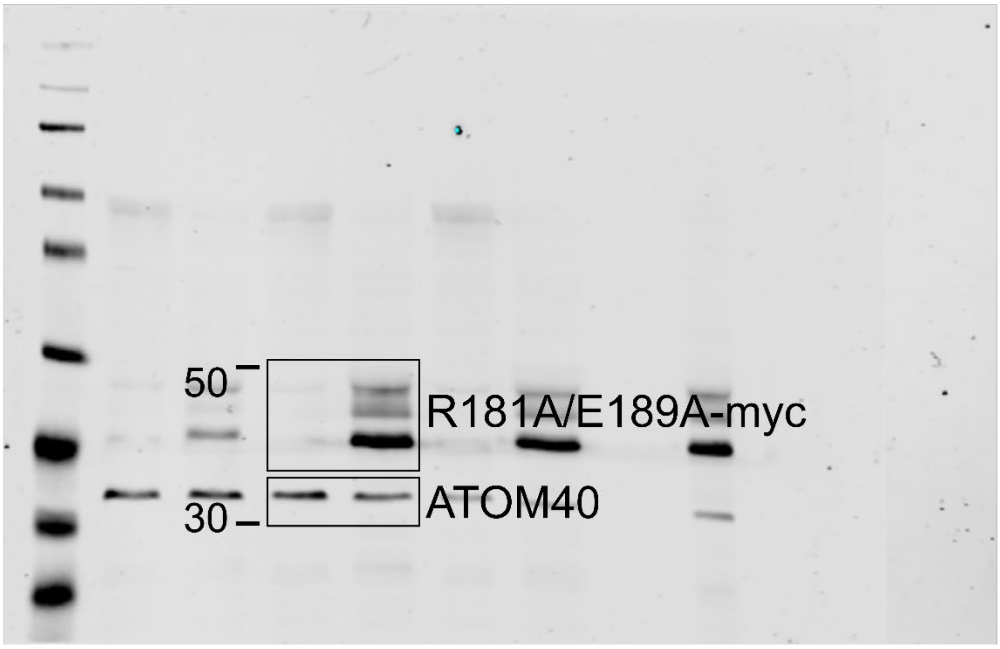

S6 Figure B middle (western blot)

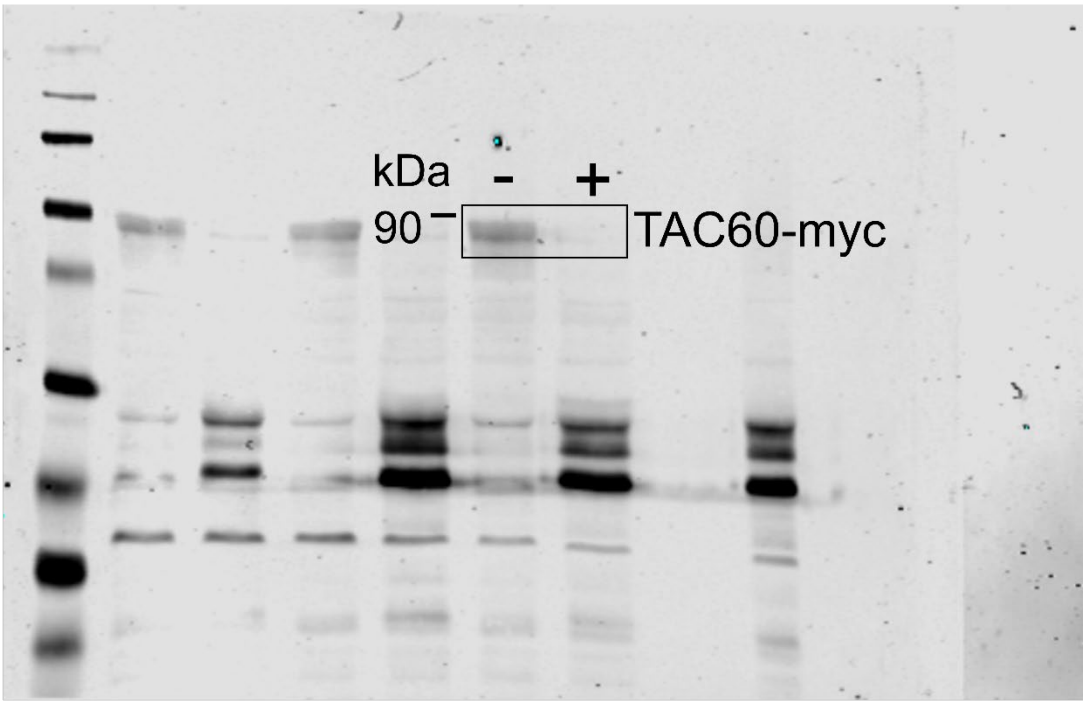

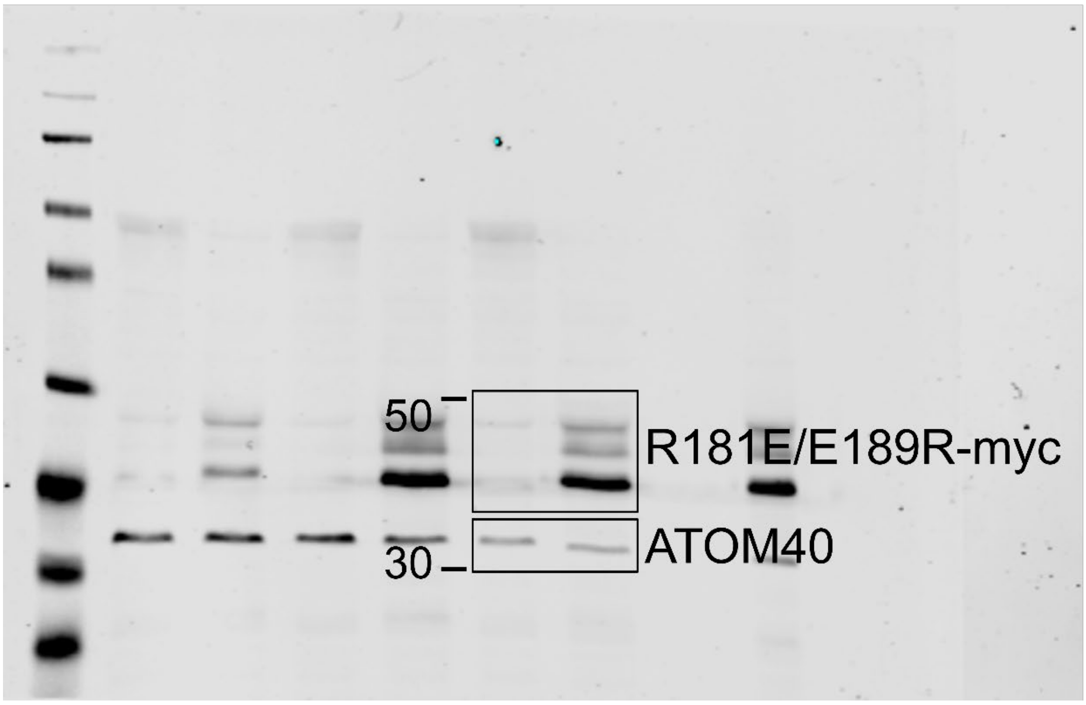

S6 Figure B right (western blot)

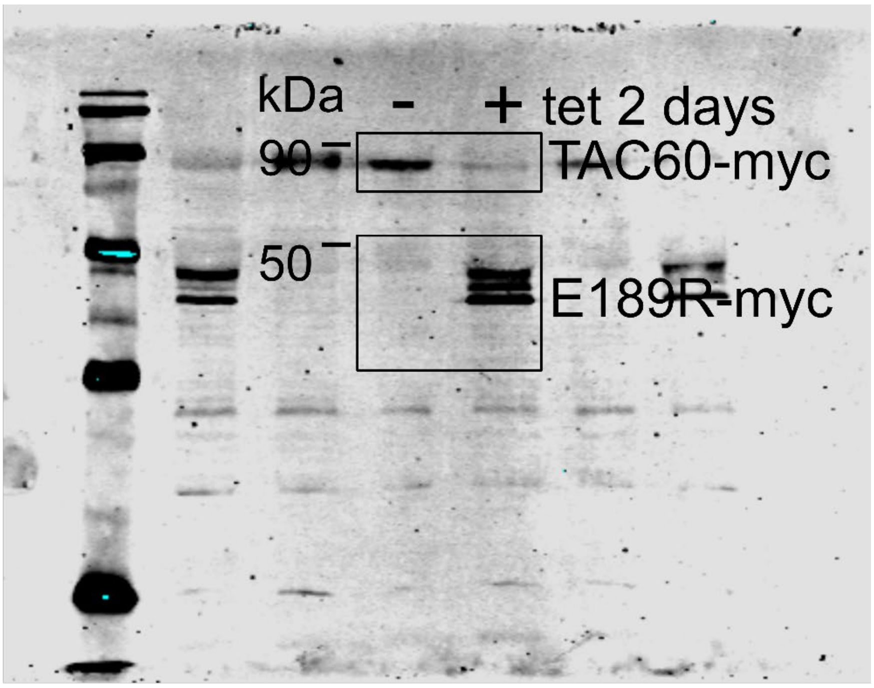

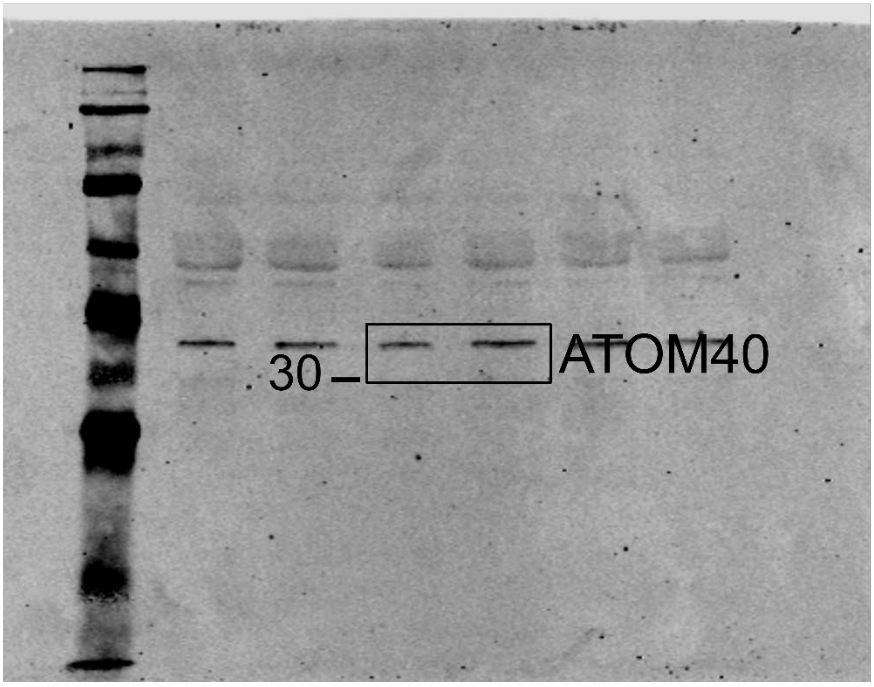

S6 Figure C left (western blot)

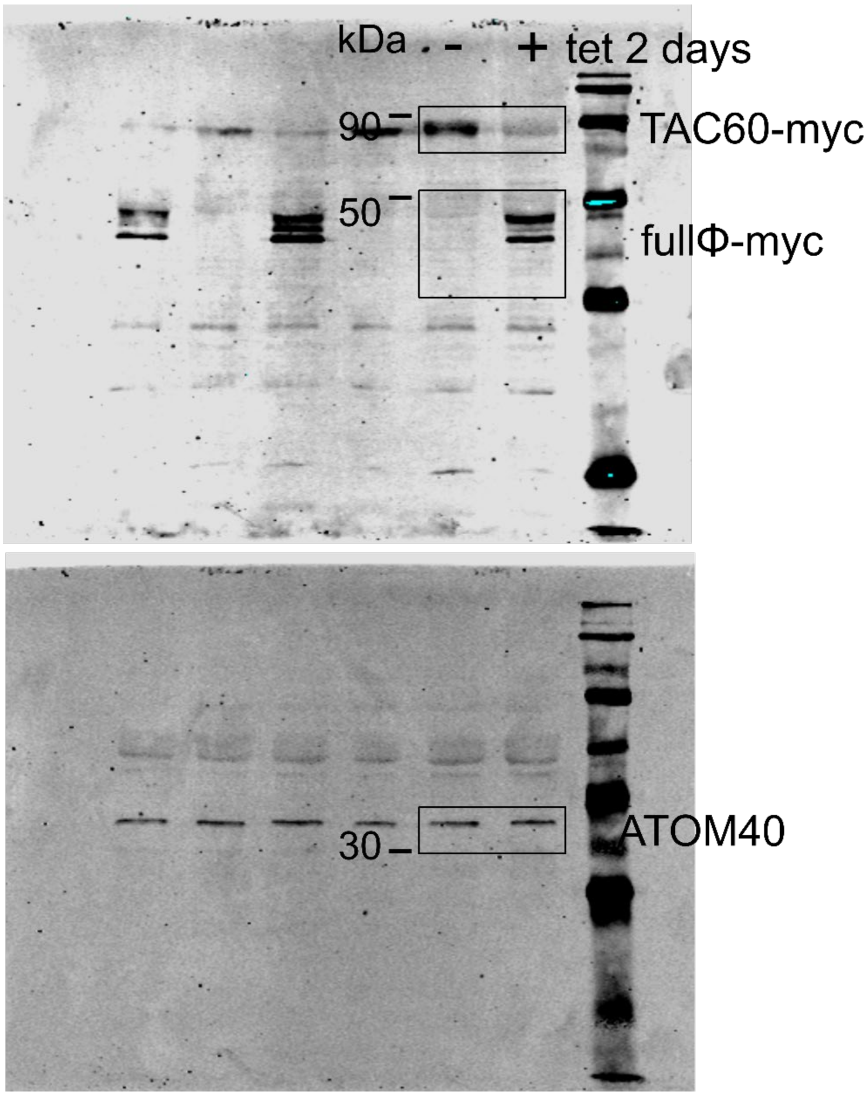

S6 Figure C right (western blot)

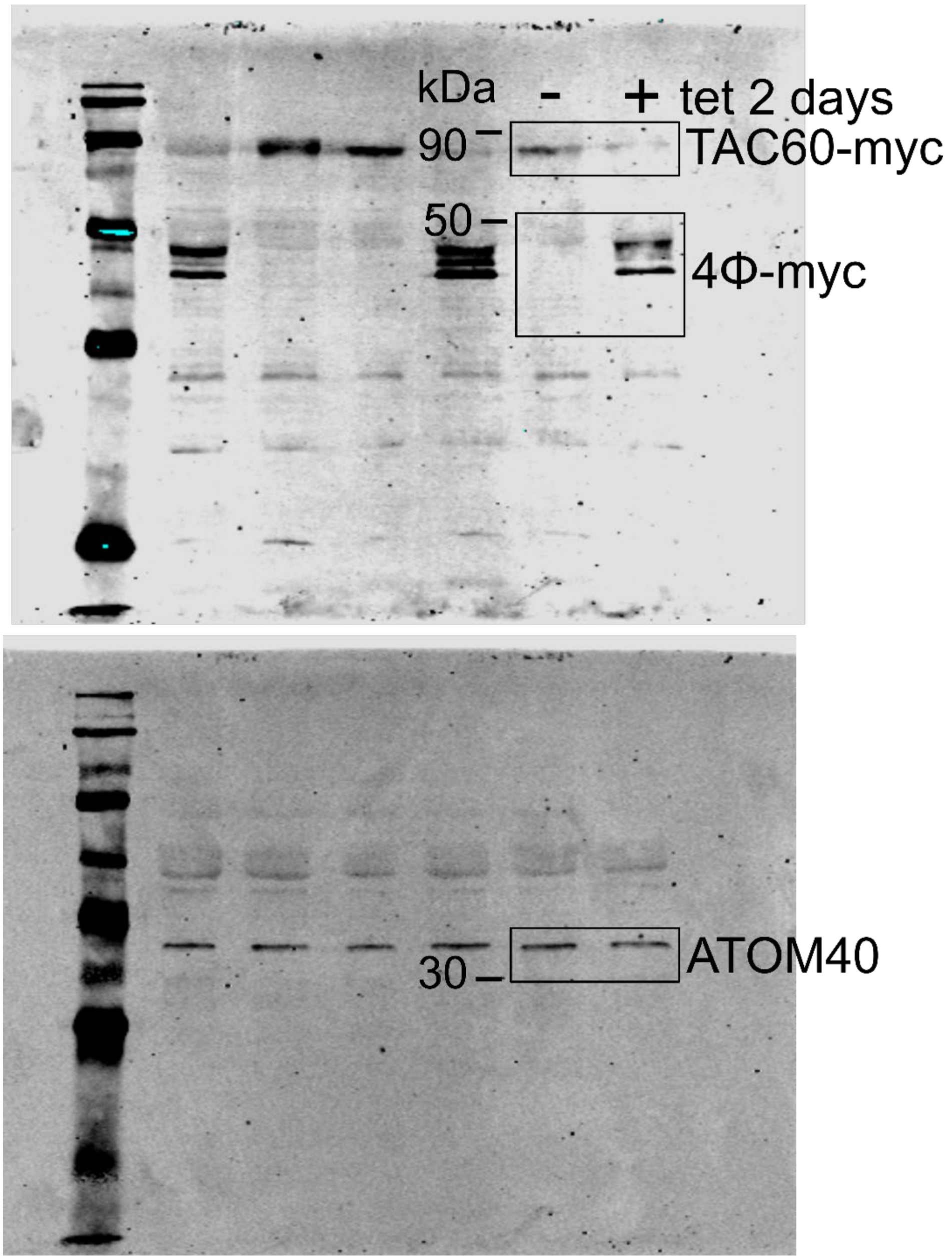

Supplement: S1 Raw images — (PDF) [file ppat.1012635.s010.pdf]
