# Supplementary material for: Molecular characterization of the permanent outer-inner membrane contact site of the mitochondrial genome segregation complex in trypanosomes
Source: PLoS Pathog. 2024 Dec 2;20(12):e1012635. doi: 10.1371/journal.ppat.1012635 (PMC11637284; doi:10.1371/journal.ppat.1012635)
Supplement: Attachment — Submitted filename: Stettler-24-resp-to-rev.pdf [file ppat.1012635.s011.pdf]

**Part I - Summary**

***Reviewer #1:***

*Stettler et al. conducted a comprehensive investigation into the molecular interactions between the outer membrane protein TAC60 and the inner membrane protein p166 within the tripartite attachment complex (TAC) of Trypanosoma brucei. The TAC is a crucial structure that links the basal body of the flagellum to the kinetoplast, playing a key role in the proper segregation of mitochondrial DNA during cell division in this protozoan parasite.*
*By employing a combination of computational predictions and experimental approaches—both in vitro and in vivo—the authors identified specific protein regions and amino acids essential for the interaction between p166 and TAC60. Their analysis narrowed down the critical elements to hydrophobic side-chain contacts between a kinked α-helix in TAC60 and the C-terminal α-helix of p166. This detailed insight into the protein-protein interface advances our understanding of the structural basis for TAC function.*
*The study not only extends existing knowledge about the TAC but also provides important molecular details of the interaction between its components. The high-quality data, derived from rigorous experimental design and execution, lend strong support to the authors' conclusions. The narrative is well-written and effectively communicates the most salient points, guiding the reader through the complex methodologies and findings with clarity.*
*While the objective of the study is somewhat narrowly focused, its implications are significant. By elucidating the specific contacts critical for TAC assembly and function, the research offers valuable information that could inform future studies on kinetoplast segregation and flagellar attachment. This could have broader impacts on our understanding of mitochondrial inheritance and the biology of Trypanosoma brucei.*
*In conclusion, Stettler et al. have made a noteworthy contribution and their work enhances our understanding of the TAC and sets the stage for future investigations into the mechanisms of mitochondrial DNA segregation in eukaryotes.*

Many thanks for this positive review

***Reviewer #2: Purpose of the study:***

*The authors aimed to investigate the molecular interface of the TAC60 and p166 interaction and its role in mitochondrial genome maintenance. Trypanosomes have a single copy mtDNA, known as kDNA, anchored to the basal body, via the essential tripartite attachment complex (TAC). This physical linkage enables co-segregation of the mtDNA along with the basal body during cell division. TAC is comprised of many multi-protein modules, with p197 linking the basal body to pATOM36 and TAC60 at the mitochondrial outer membrane. Previous studies have shown that p166, a mitochondrial matrix protein is essential for kDNA biogenesis (Zhao et al., 2008). Recently, a study from the authors group showed that p166 has a transmembrane domain, localizes to the IM and consists of a C-terminal tail that extends to the IMS, thereby linking the TAC to kDNA (Schimanski et al., 2022). However, the molecular*

*architecture of the TAC60-p166 interaction at the IM reamins unclear.*

*Brief Summary:*
*In this study, the authors employ in silico, in vitro and in vivo methods to establish the minimum-binding region of TAC60 that interacts with p166. They validate AlphaFold predictions with in vitro peptide binding assay and phylogenetic analyses. In vivo pull down of TAC60 and its variants show that a helical kink in TAC60 and conserved hydrobhobic amino acids in the interface are necessary for the TAC60-p166 interaction and kDNA maintenance. The study provides significantly novel and very interesting data leading to detailed insights into a molecular understanding of the assembly of the mitochondrial genome segregation complex in trypanosomes.*

*The experiments are of high quality and appropriately controlled.*
*The manuscript can in principle be published as it is.*
*I have only a very few comments which the authors might find usefull to follow in a revised version of the manuscript:*

*Since the TAC60-p166 acts as a permanent physical linkage between the basal body and kDNA, it would be interesting to determine (or at least estimate) the binding affinity. The recombinant p166 C-tail from FigS2 could be tested for its binding with the various domains of TAC60 and mutants by isothermal calorimetry or similar methods. From their previous study (Schimanski et al., 2022), immunoprecipitation of mini-p166-HA can pulldown multiple TAC components. Hence, in vivo pulldowns cannot rule out indirect interactions. In vitro binding assays would provide more clarity to the strength of binding between TAC60 and p166.*

We agree it would be interesting to know the binding affinity. However, taking into account the numerous mutants we tested, we believe this is beyond the scope of the present study.

*It could be a good control to analyze the TAC complexes in mitochondria isolated from TAC60 and p166 mutant cells in comparison to the controls on Blue-Native PAGE gels whether the interactions that they observed in pulldowns form a native protein complex or how the native TAC complex behaves.*

This is a difficult experiment. As mentioned in the manuscript the full length p166 present in the native TAC is essentially insoluble and thus cannot be resolved by BN-PAGE. This is the reason why we used the mini-p166.

*In Line 212, it should be specified Fig.4C, middle panel.*

The phrase refers to growth inhibition. Thus it should refer to left panel, which shows the growth curve. This has been corrected in the revised manuscript.

*In Line 231, the authors state that Mini-p166-HA was completely recovered into the SN2 fraction referring to Fig. 4D, however there is a considerable amount of Mini-p166-HA*

*present in the FT fraction, the text should be corrected as Mini-p166-HA was predominantly or mainly recovered into the SN2 fraction.*

The statement in the manuscript is correct: Mini-p166-HA was essentially completely recovered into the SN2 fraction. It is also true that an estimated 50% of mini-p166-HA is found in the FT fraction. This is not a contradiction, since the SN2 fraction corresponds to the sum of the FT and the IP fractions.

*In Figure 4C, can the authors explain why three different bands are observed in ΔC283-myc immunoblot membrane?*

We quite consistently observed two to three bands for TAC60 and its variants (depending on the gel) although how much of each bands is present can be variable in the different fractions. Presently we do not know what the cause of these multiple bands is. All bands are detected by anti tag antibodies meaning that all detected TAC60 variants have an intact C-terminus. Thus the multiple bands could in principle be due to partial N-terminal degradation although essentially the same pattern is also observed for N-terminal truncated variants [1]. - We have some preliminary evidence that for the full length TAC60 phosphatase treatment simplified the banding pattern and shifts it towards a lower molecular weight range. Thus, the observed heterogeneity within the TAC60 variants might be caused by phosphorylation and possibly other postranslational modifications. – The multiple bands are now discussed in the revised manuscript (lines 284-287).

1. Käser S, Willemin M, Schnarwiler F, Schimanski B, Poveda-Huertes D, Oeljeklaus S, et al. Biogenesis of the mitochondrial DNA inheritance machinery in the mitochondrial outer membrane of Trypanosoma brucei. PLoS Pathog. 2017;13(12):e1006808. doi: 10.1371/journal.ppat.1006808. PubMed PMID: 29287109.

*In Figure 4 B and C, western blot analyses could be shown in separate panels instead of illustrating them on the graph as such small figures.*

We modified the Figures, the immunoblots are now larger.

*In Figures 4D and in Figures 5,6,7, in pulldown assays, TAC40 immunoblot membrane is cut quite close to the signal, the authors could cut the signal with more space around the edges, if it is possible.*

The signal was on purpose cut quite narrow because the blots were reprobed for ATOM40 which runs very close to TAC40. - Moreover the heavy chain of the antibody which that leaked from the beads was also detected by the TAC40 antiserum. – A full scan of all gels and immunoblot is now provided as “S1 Raw Images”.

*In Figures 4B, 4C, 5A, 5B, 5C a.u. abbreviation should be described as arbitrary units in the figure legends as well as in other diagrams of DAPI stained kDNA area measurements.*

Done

*In Figures 5A, 7E and 8A, in pulldown assays, TAC40 signal in the bound fraction (IP) runs higher than the other lanes. Can the authors explain the reason?*

The reviewer is correct but this concerns the entire lanes, not just the TAC40 signal but also the tested TAC60 variant runs a bit higher in the indicated figures. This is likely due to the fact that in the bound fraction, which corresponds to the eluate of the whole immunoprecipitation, the loaded sample volume was the highest. This caused a slightly different migration than is observed in the other samples.

***Reviewer #3:***

*This study offers a detailed exploration of the molecular framework involved in mitochondrial genome segregation in Trypanosoma brucei, specifically focusing on the role of the tripartite attachment complex (TAC). The TAC serves as a physical link between the mitochondrial genome and the flagellum basal body, with two proteins, TAC60, located in the outer membrane, and p166, in the inner membrane, playing central roles. Through a combination of computational modelling, mutational analysis, in vitro and in vivo assays, the researchers mapped the interaction between these proteins.*

*One of the study's key revelations is the identification of a short kinked α-helix in TAC60 that binds to the C-terminal α-helix of p166. Interestingly, this interaction is driven by hydrophobic forces rather than the more common electrostatic interactions, highlighting the importance of hydrophobic residues in maintaining this critical link. By pinpointing the minimal binding regions and emphasising the significance of hydrophobic interactions, the research deepens our understanding of how the TAC operates at a molecular level to ensure proper mitochondrial genome segregation. The study adds valuable insights into the structural organisation of the TAC and also opens avenues for further research into potential therapeutic targets within this excessively complex system.*

*The manuscript is mainly easy to read, well-written and clear, with good-quality data. It has a well-argued analysis of the problem and comprehensible and concise results. It is sometimes a little tricky to follow. It only requires one major modification and a few minor ones, and if done, in my opinion, it would make it acceptable for publication in PLoS Pathogens.*

**Part II – Major Issues: Key Experiments Required for Acceptance**

***Reviewer #1:*** *None.*

***Reviewer #2:*** *(No Response)*

**Reviewer #3:** *Major comments*

*An obvious question raised from reviewing this work is: can TAC60 proteins from other species bind to the w/t of T. brucei's proteins P166? And, can p166 from other species*

*bind to the w/t of T. brucei's TAC60? It would be interesting to test this, focusing on genes from T. cruzi and a Leishmania species (closely or distantly related). Having asked the questions, I realize it is perhaps beyond the scope of this current work to do this in the lab, but in silico, it is possible. An alpha-fold2 analysis to address these two questions, albeit limited, would be relatively simple and easy to do (Similar to Fig1C).*

The suggested experimental analyses would be very labour intensive. Moreover, we are not convinved that having this information, either as experimental results or as in silico analyses, would be very informative. It represents by definition a non physiological situation, because in vivo TAC60 and p166 within each species have been adapted to each other by coevolution. – However, knowing to which extent the structures of the TAC60-p166 interface and their predicted mode of interaction are conserved in different kinetoplastid species would indeed be interesting. Thus we decided to add the additional supplementary Fig. S7 which shows the structural prediction of TAC60-p166 interactions for.....

---

**Part III – Minor Issues: Editorial and Data Presentation Modifications**

***Reviewer #1:*** None.
***Reviewer #2:*** (No Response)
***Reviewer #3:*** Minor comments

*1. Figure S6, in C, the alignment of the IFA signals on the TAC60 mutants is misaligned and needs to be checked.*

The Figure has been corrected accordingly

*2. The authors used the monoclonal antibody YL1/2 to probe and locate basal bodies, and they stated that this is "Tyrosinated tubulin, detected by YL1/2". YL1/2 indeed binds tyrosinated tubulin, but it has been shown that in trypanosome basal bodies, YL1/2 detects TbRP2, ref, (Volume 168, Issue 4, August 2017, Pages 452-466). For clarity, the authors should note this in their manuscript.*

This information and the citation has been added to the revised manuscript

*3. The methods section has page and alignment-related problems.*

The font size was different. This has been corrected.

*4. What happened is unclear, but the conversion to .pdf format may have separated hyphenated words throughout the text and removed the Greek symbol for alpha*

Our PDF looks fine.
